# DNA Methylation Influences Chlorogenic Acid Biosynthesis in *Lonicera japonica* by Mediating LjbZIP8 to Regulate Phenylalanine Ammonia-Lyase 2 Expression

**DOI:** 10.3389/fpls.2017.01178

**Published:** 2017-07-10

**Authors:** Liangping Zha, Shuang Liu, Juan Liu, Chao Jiang, Shulin Yu, Yuan Yuan, Jian Yang, Yaolong Wang, Luqi Huang

**Affiliations:** ^1^College of Pharmacy, Anhui University of Chinese MedicineHefei, China; ^2^State Key Laboratory of Dao-di Herbs, National Resource Center for Chinese Materia Medica, China Academy of Chinese Medical SciencesBeijing, China; ^3^Institute of Traditional Chinese Medicine Resources Protection and Development, Anhui Academy of Chinese MedicineHefei, China; ^4^School of Chinese Materia Medica, Beijing University of Chinese MedicineBeijing, China

**Keywords:** *Lonicera japonica*, DNA methylation, bZIP, PAL, transgenic tobacco, CGAs biosynthesis

## Abstract

The content of active compounds differ in buds and flowers of *Lonicera japonica* (FLJ) and *L. japonica* var. chinensis (rFLJ). Chlorogenic acid (CGAs) were major active compounds of *L. japonica* and regarded as measurements for quality evaluation. However, little is known concerning the formation of active compounds at the molecular level. We quantified the major CGAs in FLJ and rFLJ, and found the concentrations of CGAs were higher in the buds of rFLJ than those of FLJ. Further analysis of CpG methylation of CGAs biosynthesis genes showed differences between FLJ and rFLJ in the 5′-UTR of phenylalanine ammonia-lyase 2 (*PAL2*). We identified 11 *LjbZIP* proteins and 24 *rLjbZIP* proteins with conserved basic leucine zipper domains, subcellular localization, and electrophoretic mobility shift assay showed that the transcription factor LjbZIP8 is a nuclear-localized protein that specifically binds to the G-box element of the *LjPAL2* 5′-UTR. Additionally, a transactivation assay and *LjbZIP8* overexpression in transgenic tobacco indicated that LjbZIP8 could function as a repressor of transcription. Finally, treatment with 5-azacytidine decreased the transcription level of *LjPAL2* and CGAs content in FLJ leaves. These results raise the possibility that DNA methylation might influence the recruitment of LjbZIP8, regulating *PAL2* expression level and CGAs content in *L. japonica*.

## Introduction

*Lonicera japonica* is a perennial tropical tree species, and widely used in traditional Chinese medicine, health foods, cosmetics, and as an ornamental groundcover (Shang et al., 2011). Phytochemical studies revealed that the phenolic constituents, iridoid glycosides, cerebrosides, saponins, and volatile oils are the active components responsible for the beneficial medicinal properties of FLJ (Shang et al., 2011). In particular, the phenolic constituents, namely phenolic acids and flavonoids, which are major components with a broad spectrum of antibacterial, anti-inflammatory, antioxidant, and anti-viral effects in FLJ (Shan et al., 2009; Lee et al., 2010; Jurikova et al., 2011; Seo et al., 2012). Chlorogenic acid and luteoloside are regarded as marker compounds for the quality evaluation and standardization of FLJ (Pharmacopoeia of the People’s Republic of China, 2015).

A limited number of publications have reported on the formation of active compounds in the buds and flowers of FLJ, and investigated their biosynthesis using molecular biological techniques. Yuan et al. (2014) provided a comprehensive overview of the gene families involved in CGAs biosynthesis in FLJ. The biosynthesis of CGAs was found to be controlled by the phenylpropanoid pathway, the universal precursor p-coumaroyl-CoA is catalyzed by PAL, 4CL, C4H, and HQT. CGAs content was shown to be significantly correlated with the expression level of *LjCCoAOMT1* in various tissues of *L. japonica* at different developmental stages (Jiang et al., 2014).

Active compounds content also vary significantly among species and varieties of the *Caprifoliaceae* family. *L. japonica* Thunb. var. chinensis (rFLJ) is a Chinese local variety whose corolla has purple outer (upper) and white inner (lower) portions. The rFLJ flower has different content of active compounds compared to those of FLJ (Lin et al., 2015). A systematic study of floral transcriptomes was performed to compare gene expression and variations in active compounds of different varieties. The phylogenetic tools for the analysis of paralogs revealed that the reads per kilobase of transcript per million (RPKM) values of *PAL*, *CHS*, and *HMGR* were higher in rFLJ compared with those in FLJ (Yuan et al., 2012). Therefore, variation in gene expression may account for differences in the content of active compounds between rFLJ and FLJ.

Recent studies in molecular genetics have revealed that gene expression could be affected by epigenetic mechanisms, even in the absence of genetic variation (Peredo et al., 2006). CpG (mCG) methylation at the C_5_ position of cytosine (mC) is found mostly in DNA methylation, which is a major epigenetic mechanism identified in higher plants and animals (Jin et al., 2016). This process blocks transcription factors from binding to methylated regions of a promoter, which is an effective mechanism of transcription regulation and has been demonstrated in many instances (Curradi et al., 2002).

Previous studies have shown that bZIP proteins, a group of transcription factors, play important roles in organ and tissue differentiation, energy metabolism, nitrogen/carbon, balance control, hormone and sugar signaling, and pathogen defense (Jakoby et al., 2002; Corrêa et al., 2008; Weltmeier et al., 2009; Dietrich et al., 2011; Alves et al., 2013; Lozano-Sotomayor et al., 2016). It has been reported that OsbZIP23 acts as a central regulator in ABA signaling by positively regulated the expression of *OsNCED4*, a key gene in ABA biosynthesis. Sibéril et al. (2001) reported that CrGBF1 and CrGBF2 acted as transcriptional repressors via direct interaction with the G-box associated with *strictosidine synthase* in *Catharanthus roseus*. However, there were no papers reported on the expression or function of the bZIP family members in *L. japonica*.

In this study, we sought to validate the hypothesis that the expression level of *PAL2* and content of CGAs might been influenced by methylation-mediated transcription factor regulation in *L. japonica*. First, the content of major CGAs presented in FLJ and rFLJ grown under the same environmental conditions were analyzed. Then, we analyzed the CpG methylation levels of the 5′-UTR of CGAs biosynthesis genes between FLJ and rFLJ, which revealed that the CpG methylation levels in the 5′-UTR of *PAL2* were different between FLJ and rFLJ. We further identified 11 LjbZIP proteins in FLJ and 24 rLjbZIP proteins in rFLJ, and 6 bZIP proteins were selected for EMSA analysis to show which could bind specifically to the G-box element of the *PAL2* promoter. In addition, a transactivation assay and LjbZIP8 overexpression in transgenic tobacco were performed to validate the function of LjbZIP8. In order to validate the hypothesis *in vivo*, DNA methylation inhibitor (5-aza) was performed to elucidate the methylation-mediated regulation of spatiotemporal expression of *LjPAL2* in CGAs biosynthesis and content of CGAs. These results established a complete regulatory network concerning transcription factor in the CGAs signaling pathway in *L. japonica*.

## Materials and Methods

### Plant Materials

Fresh buds of FLJ and rFLJ were collected in Spring 2013 from the Gardens of Yate Co., in Shandong Province, China. Samples were authenticated by Prof. Liying Yu (Guangxi Medical Plant Garden, China) and stored in liquid nitrogen. FLJ plants were cultivated in outdoor flowerpots near laboratory, and watered regularly to keep the soil moist. The impact of temperature and humidity on perceived air quality was studied in the ranges 20–30°C and 40–60% RH. Leaves of living FLJ plants were wiped gently with 80 μM 5-aza by cotton swabs for 24 h (about 12 h light/12 h dark in one diurnal time), while untreated leaves were used as control group. Treated and untreated leaves were from the same plants, and the experiment was repeated three times.

### Genomic DNA Isolation

Genomic DNA was isolated by the cetyl trimethyl ammonium bromide (CTAB) method as described by Doyle (1991). DNA was quantified using a NanoDrop 2000 Spectrophotometer (Thermo Scientific, San Jose, CA, United States) and stored at 4°C until further analysis.

### Bisulfite PCR Primer Designing and Bisulfite Sequencing

Bisulfite sequencing primers were designed based on the 5′-UTR sequences using MethPrimer (Li and Dahiya, 2002). The optimum product size (<200 bp), Tm (<55°C), and primer size (<25 bp) were taken into consideration for primer design. Primers were designed to flank the CG-rich regions of the promoter sequence. All primers for bisulfite sequencing were designed avoiding CpGs in the sequence. Conventional primers were also designed using Primer3^1^ for amplification of the respective 5′-UTR regions of untreated DNA that served as the master sequence for bisulfite sequence analysis. Bisulfite DNA conversion was performed using 1 μg of genomic DNA and an EpiTech Bisulfite Kit (Qiagen, Hilden, Germany), following the manufacturer’s protocol. PCR was performed using primers located outside the target region and designed for single-strand methylation detection. PCR products were cloned into pCR2.1 using a TA cloning kit (Invitrogen, Carlsbad, CA, United States). For each genotype, at least 20 independent clones were sequenced using the M13R primer, and data were analyzed by Cymate (Hetzl et al., 2007).

### Quantitative DNA Methylation Analysis

The methylation levels of selected genes were detected as described by Shinozuka et al. (2015). Briefly, 100 ng of genomic DNA was subjected to two separate treatments: methylation-sensitive digestion and mock digestion. For the methylation-sensitive digestion, the methylated DNA was digested by the methylation-sensitive enzyme *Msp*JI, while the remaining un-methylated DNA was detected by RT-PCR. For the mock digestion, inactive *Msp*JI enzymes were added to the reaction and the products represented the total amount of input DNA. The relative amount of each DNA fraction (methylated and un-methylated) was calculated by using the standard ΔCt method (Ordway et al., 2006), normalizing the amount of DNA in each digestion against the total amount of input DNA in the mock digestion. The amount of methylated DNA was defined as 2^-Ct(Mo-Ms)^/2^-CtMo^, where 2^-CtMo^ is the total amount of input DNA, and 2^-Ct^ ^Ms^ is the amount of un-methylated DNA.

Genomic DNA was divided into two parallel groups for *Msp*JI digestion or mock digestion. The *Msp*JI digestion solution included 100 ng of genomic DNA, 10 units of *Msp*JI (New England Biolabs, Ipswich, MA, United States), 1 × NEBuffer 4 (New England Biolabs, Ipswich, MA, United States), 0.5 μM reaction activator, and deionized water to a final volume of 20 μL. The mock digestion solution included inactive *Msp*JI enzymes. Reactions were incubated at 37°C for 4 h, and followed by heat inactivation at 65°C for 15 min.

The RT-PCR solution included 10 μL 2 × I-5^TM^ High-Fidelity Master Mix (MLAB, Los Angeles, CA, United States), 0.4 μL 50 × ROX (Applied Biosystems, Foster City, CA, United States), 0.4 μL forward and reverse primers (10 μM), 1 μL 20 × EvaGreen (Biotium, Hayward, CA, United States), and 2 μL reaction temple (endonuclease-treated samples diluted 1:10 with double-deionized water). Primer sequences are listed in Supplementary Table S1. RT-PCR conditions were as follows: 2 min at 50°C, 3 min at 95°C, followed by 40 cycles of 10 s at 95°C and 20 s at 58°C. Melting curves were used to verify the specificity of PCR products by increasing the temperature from 60°C to 95°C in sequential steps of 0.2°C for 15 s. Amplification was performed in triplicate, and the no-template control was performed for each gene. The relative abundance of genes was determined using the comparative Ct method in ABI 7500 2.0.1 (Applied Biosystems, Foster City, CA, United States).

### Regulatory Motif Search

The current assembly of FLJ genomic sequences was investigated for the 5′-UTR region of *LjPAL1* (JX068601), *LjPAL2* (JX068602), *LjPAL3* (JX068603), *Lj4CL1* (JX068604), *Lj4CL2* (JX068605), *LjC4H1* (JX068606), *LjC4H2* (JX068607), and *LjHQT* (ACZ52698) using the Blastn algorithm (Altschul et al., 1997). An *e*-value cut-off of 10^-30^ was applied for homolog recognition. The obtained 5′-UTR regions were subjected to regulatory sequence search analysis using PlantCARE and Softberry to identify the *cis*-regulatory elements in each sequence (Lescot et al., 2002).

### Identification of LjbZIP Proteins in FLJ

We applied RNA-seq to FLJ and rFLJ and generated over 100 million reads using the Illumina GA platform (Yuan et al., 2012). The current assembly of FLJ transcriptome was investigated for bZIP homologs in *Arabidopsis thaliana* using the BLASTx algorithm (Altschul et al., 1997). An *e*-value cut-off of 10^-5^ was applied for homolog recognition. The rFLJ transcriptome was searched for orthologs from FLJ genes. An all-against-all sequence comparison was performed with BLAST (cut-off < 10^-20^) to identify orthologs, which were determined from the best reciprocal hits (80% alignment length) as described by Tatusov et al. (1997). All retrieved sequences from Genscan^2^ were used for gene prediction (Burge and Karlin, 1998). The predicted gene models were further examined and corrected by manually comparing them with related genes of other plant species. The functional and structural domains were predicted by InterProScan (Zdobnov and Apweiler, 2001) and Blast2GO (Conesa et al., 2005), respectively.

### Construction of Phylogenetic Trees

The deduced amino acid sequences were adjusted manually by BioEdit 7.0.0^3^ using the default parameters. The ORF of *LjbZIP*s was identified by BioEdit (Hall, 1999). The phylogenetic analysis of alignments was performed by ClustalW (Thompson et al., 2002) and MEGA 6.0 (Tamura et al., 2013) using neighbor-joining analysis. The reliability of tree topologies was evaluated using bootstrap support with 1,000 replicates (Pattengale et al., 2010)^.^ The sequences of 57 *Arabidopsis bZIP*s were downloaded from the *Arabidopsis Information Resource*^4^.

### Recombinant Expression and Purification of LjbZIPs in *E. coli*

*LjbZIP11* (Group A), *rLjbZIP4* (Group A), *LjbZIP8* (Group C), *rLjbZIP18* (Group C), *LjbZIP10* (Group D), and *rLjbZIP1*(Group D) cDNAs were amplified by PCR and cloned into pGEM T-easy cloning vector. After confirming inserted sequences, the cDNAs were double-digested with *Bam*HI and *Sal*I, and inserted into the pGEX-4T-1 expression vector that had been digested with *Bam*HI and *Sal*I. The recombinant plasmid pGEX-LjbZIP11, pGEX-rLjbZIP4, pGEX-LjbZIP8, pGEX-rLjbZIP18, pGEX-LjbZIP10, and pGEX-rLjbZIP1were transformed into *Escherichia coli* BL21 (DE3) chemically competent cells (Beijing TransGen Biotech, Beijing, China). Expression of the recombinant protein was induced with 0.4 mM IPTG at 16°C overnight, and cells were resuspended in PBS buffer (137 mmol/L NaCl, 2.7 mmol/L KCl, 10 mmol/L Na_2_HPO_4_, and 2 mmol/L KH_2_PO_4_) and disrupted by sonication. The recombinant protein were purified by affinity chromatography by using *ProteinIso* GST resin (TransGen Biotech, Beijing, China) following the manufacturer’s instruction. The purified lysate was centrifuged at 12,000 × *g* for 30 min at 4°C, and the supernatant was loaded onto a 8% SDS–PAGE gel after denaturation with SDS loading dye at 100°C for 5 min. The gel was stained with Coomassie Brilliant Blue G-250 and decolorized with a destaining solution (70% Ultrapure water; 10% ethanol; 20% acetic acid) (Zha et al., 2016).

### Electrophoretic Mobility Shift Assay

The G-box sequence in the promoter sequence of *LjPAL2* (GenBank: JX068602) was obtained using Softberry. The oligonucleotides (5′-CTCTCCTGTCCACGT GTCATCAAC-3′ for the G-box sequence) were synthesized and labeled with biotin (Sangon Biotech, Shanghai, China) using the Lightshift Chemiluminescent EMSA kit (20148, Pierce, Thermo Fisher Scientific, United States). Complementary labeled strands were mixed together in an equimolar ratio and annealed at 25°C after denaturation at 90°C. Gel mobility shift assays were performed by incubating 40 fmol of labeled probe with 15 fmol LjbZIP proteins (LjbZIP11, LjbZIP8, LjbZIP10, rLjbZIP1, rLjbZIP4, and rLjbZIP18) and 4 pmol (or 0 pmol) competing oligonucleotides in the binding reactions at room temperature for 20 min. Mixtures were size fractionated in a non-denaturing 30% polyacrylamide gel followed by drying, transferred to nitrocellulose membranes, and detected by streptavidin-HRP/chemiluminescence for biotin-labeled probes.

### Subcellular Localization

The *LjbZIP8* sequence was ligated into the pE3025 vector, which was digested with *Eco*RI and *Kpn*I to generate pGEM-LjbZIP8 plasmids. *LjbZIP-GFP* was under the control of the CaMV 35S promoter. The construct was confirmed by sequencing and used for the transient particle-bombardment transformation of onion (*Allium cepa*) epidermis cells using a gene gun (Bio-Rad, Hercules, CA, United States). Following 24 h of post-bombardment incubation, GFP fluorescence in transformed onion cells was observed under a confocal microscope (Zeiss, Oberkochen, Germany).

### Transactivation Assay

To determine the transactivation activity, the ORF of *LjbZIP8* was generated by PCR amplification and cloned into the *Eco*RI/*Sal*I digested pGBKT7 vector to generate pBD-LjbZIP8 plasmids. The constructs were transformed into YGR2 cells by the lithium acetate-mediated method (Yuan et al., 2013). The transformed yeast strains were placed on SD/–Trp medium at 28°C for 2 days. Yeast transformants were then transferred and streaked onto solid SD/–Trp/–His/–Ade to score the growth response after 3 days. The pBD-GAL4 and pGBKT7 vectors were used as positive and negative controls, respectively.

### Tobacco Transformation

*LjbZIP8* was inserted into the binary vector pCambia1305 to produce p35Spro-LjbZIP8 plasmids that were then used to transform *Agrobacterium tumefaciens* EHA105. Tobacco (*Nicotiana tabacum*) leaf disks were transformed via an *A. tumefaciens* mediated leaf disk procedure (Horsch et al., 1989) and selected using 50 mg/L Hygromycin B and 200 mg/L carbenicillin. After rooting and acclimatization, the regenerated plants were cultivated in a greenhouse to set seeds by self-pollination. Transgenic plants were used for further analysis.

### Quantitative Real-Time PCR

Total RNA was reverse-transcribed using Reverse Transcriptase MMLV (Takara, Dalian, China). PCR was performed in triplicate using an ABI 7500 Real-Time PCR System (Applied Biosystems, Foster City, CA, United States) with SYBR Premix Ex Taq kit (TaKaRa, Dalian, China), according the manufacturer’s instructions. Gene-specific primers of *NtPAL1* (M84466), *NtPAL2* (D17467), *NtPAL4* (EU883670.1), and *LjbZIP8* (KT218632) were designed using Primer3 (Supplementary Table S1). The length of PCR products ranged from 100 to 250 bp. *Ntactin* was chosen as an endogenous control for studying gene expression in various samples of transgenic tobacco. The specificity of amplification was assessed by melting curve analysis, and the relative abundance of genes was determined using the comparative Ct method as suggested by ABI 7500 2.0.1 (Applied Biosystems, Foster City, CA, United States). Significance in relative expression levels of genes were determined by a paired-samples *t*-test. *P*-values below 0.05 were considered statistically significant for all tests.

### Chemical Reagents Preparation of Sample Solution

HPLC-grade acetonitrile and methanol were obtained from Thermo Fisher Scientific (Waltham, MA, United States). Phosphoric acid was purchased from Aladdin Chemistry (Shanghai, China). Reference standards (purity > 98%), including neochlorogenic acid, CGAs, cryptochlorogenic acid, isochlorogenic acid B, isochlorogenic acid A, and isochlorogenic acid C were obtained from Tongtian Biological Technology (Shanghai, China). Deionized water was produced by Milli-Q system (Bedford, MA, United States).

Approximately 0.5 g pulverized plant samples were weighed and mixed with 50 ml of 50% MeOH aqueous solution (v/v) in a flask. The mixture was extracted in an ultrasonic bath (KQ-250DB, Kunshan Ultrasonic Instrument, Kunshan, China) for 30 min. The extraction solution was centrifuged at 4,000 rpm for 10 min, and the supernatant was diluted to 50 ml with 50% MeOH aqueous solution (v/v) and stored at 4°C. After filtration through a 0.22 μm PTFE membrane (Jinteng Technologies, Tianjin, China), the extract was ready for UPLC analysis.

### UPLC Analysis and Quantification

Chromatographic analysis was performed using an Acquity UPLC I-Class system (Waters, Milford, MA, United States) equipped with a HSS T3 column (100 mm × 1.0 mm, particle size 1.8 mm; Waters, Milford, MA, United States). The flow rate was 0.4 mL/min, and the mobile phase consisted of 0.2% phosphoric acid in water (solution A) and acetonitrile (solution B). The elution scheme was as follows: 0–2 min, 5–10% B; 2–8 min, 10–30% B; 8–11 min, 30–45% B; 11–14 min, 45–80% B; and 14–17 min, 5% B to equilibrate the column for the next injection. The column temperature was maintained at 40°C and the injection volume was 1.0 μL. Photodiode array detector spectra were measured over a wavelength range of 210–400 nm, and chromatographic data were collected and manipulated using Empower^TM^ 2 (Waters, Milford, MA, United States).

All of the CGAs were quantified using chromatograms extracted at 340 nm (Supplementary Figure S8). The content of CGAs constituents were routinely quantified using standard curves of corresponding compounds. The calibration curves were constructed using seven different concentrations (0.1, 0.5, 10, 50, 100, 500, and 1000 μL/ml) of each standard and by plotting the concentration of the standard against the peak area. The areas of overlapped peaks were calculated by UPLC.

## Results

### The Concentration of CGAs Is Higher in the Buds of rFLJ

To investigate whether the content of CGAs were different between FLJ and rFLJ, we performed analysis on different tissues by UPLC. It was observed that FLJ had lower content of neochlorogenic acid, CGAs, cryptochlorogenic acid, isochlorogenic acid B, isochlorogenic acid A, and isochlorogenic acid C compared with those in rFLJ (**Table 1**).

**Table 1:** Content of CGAs components in rFLJ and FLJ.

		Content of phenolic components (mg/g)		
No.	Analyte	rFLJ	FLJ	C_rFLJ_/ C_FLJ_
1	Neochlorogenic acid	0.55 ± 0.04	0.46 ± 0.03^∗∗^	1.20
2	Chlorogenic acid	26.67 ± 0.63	25.98 ± 0.52^∗∗^	1.03
3	Cryptochlorogenic acid	0.96 ± 0.04	0.57 ± 0.03^∗∗^	1.68
4	Isochlorogenic acid B	0.67 ± 0.04	0.30 ± 0.03^∗∗^	2.23
5	Isochlorogenic acid A	13.20 ± 0.41	11.21 ± 0.39^∗∗∗^	1.18
6	Isochlorogenic acid C	0.99 ± 0.04	0.90 ± 0.03	1.10

*The values shown are the mean ± standard deviation of three replications. The correlation coefficients are marked with ^∗^, ^∗∗^, and ^∗∗∗^ for the significance levels of 0.05, 0.01, and 0.001, respectively*.

### The CpG Methylation of 5′-UTR

Many genes (*PAL1*, *PAL2*, *PAL3*, *4CL1*, *4CL2*, *C4H1*, *C4H2*, and *HQT*) are involved in CGAs biosynthesis. Between FLJ and rFLJ, SNPs were identified in the genes involved in CGAs biosynthesis, but these did not result in changes in the amino acid sequences of these key enzymes (unpublished data). To acquire CpG island regions and candidate CpG loci, the genomic sequences of the 5′-UTR of genes involved in CGAs biosynthesis were obtained from the FLJ genome database (unpublished) and submitted to MethPrimer^5^. The results showed that 24 and 20 candidate CpG loci were located in the 452 bp 5′-UTR region of *LjPAL2* and *LjC4H1*, respectively (Supplementary Table S2 and Figure S1). However, the methylation status investigated by bisulfite sequencing PCR (BSP) indicated that DNA methylation only occurred in the 5′-UTR of *LjPAL2*. The CpG methylation ratio of *PAL2* in rFLJ (80.45%) was significantly different compared with that of rFLJ (61.24%) (Supplementary Table S3). Moreover, the expression of *PAL2* orthologs in rFLJ buds was 3.25-fold higher than that in FLJ buds (Supplementary Figure S2), which was consistent with the results in our previous study (Yuan et al., 2012).

### Identification of bZIP Proteins in FLJ

Due to DNA methylation occurs in the upstream region of *LjPAL2*, we predicted the transcription factor binding sites and regulatory elements using Softberry^6^, G-box regulatory elements were found mainly in the 5′-UTR region of *LjPAL2*. Further analysis of the transcription factors bound to the G-box sequence identified the bZIP family (Supplementary Figure S3 and Table S4). Therefore, we speculated that bZIP transcription factors might bind to the G-box of *LjPAL2*. To identify bZIP proteins in FLJ, a preliminary BLASTX search was performed against the transcriptome database. Only hits with *E*-values below e^-30^ were considered as members of this gene family. A total of 11 LjbZIP proteins and 24 rLjbZIP proteins were identified to have conserved bZIP domains, and the 11 *LjbZIP* sequences were submitted to GenBank with the accession numbers KT218625–KT218635 (Supplementary Table S5).

**FIGURE 1:**
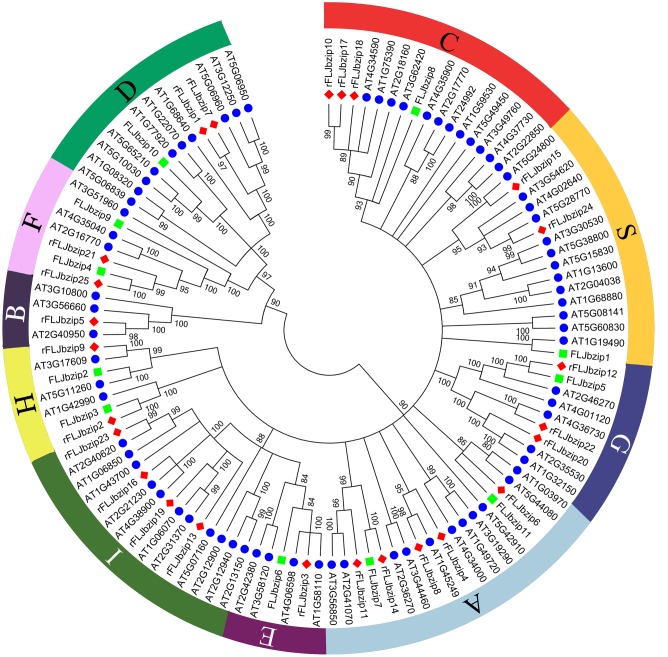
Neighbor-joining tree representing relationships among bZIP proteins from FLJ, *L. japonica* Thunb. var. chinensis (rFLJ) and *Arabidopsis thaliana* (AT). The Neighbor-Joining phylogenetic trees were constructed using the bootstrap method on MEGA 6.0 and the number of bootstrap replications was 1000. The proteins are clustered into 10 subgroups.

Based on sequence similarity, the identified *LjbZIP* sequences were clustered into 10 subgroups, according to clades with at least 80% bootstrap support (**Figure 1**). The validity of the phylogenetic reconstruction was confirmed by the fact that the subgroups were the same as those observed in previously constructed phylogenetic trees (Jakoby et al., 2002). *LjbZIP1* was classified into subgroup S, which includes homologs that are transcriptionally activated by stress (e.g., cold, drought, and anaerobic conditions, and wounding) (Kusano et al., 1995) or are expressed in specific parts of the flower (Strathmann et al., 2001). *LjbZIP2* and *LjbZIP3* were classified into subgroup H, while *LjbZIP8* belongs to subgroup C. *LjbZIP4* and *LjbZIP9* were classified into subgroup F along with *At4g35040*, *At2g16770*, and *At3g51960*. *LjbZIP5* was classified into subgroup G, which includes *GBF*s from *A. thaliana* and their parsley homologs *CPRF1*, *CPRF3*, *CPRF4a*, and *CPRF5* that are mainly linked to ultraviolet and blue-light signal transduction and the regulation of light-responsive promoters (Kircher et al., 1998). *LjbZIP7* and *LjbZIP11* were classified into subgroup A, which includes genes that play roles in ABA or stress signaling (Lescot et al., 2002). *LjbZIP10* was classified into subgroup D, which includes genes that participate in defense against pathogens (Xiang et al., 1997) and control the number of floral organs (Chuang et al., 1999). In general, the gene functions of a clade are not conserved across plant species; thus, the gene function facilitates the confirmation of paralog and ortholog relationships.

### LjbZIP8 Binds to the G-Box Element of the *LjPAL2* 5′-UTR *In Vitro*

**FIGURE 2:**
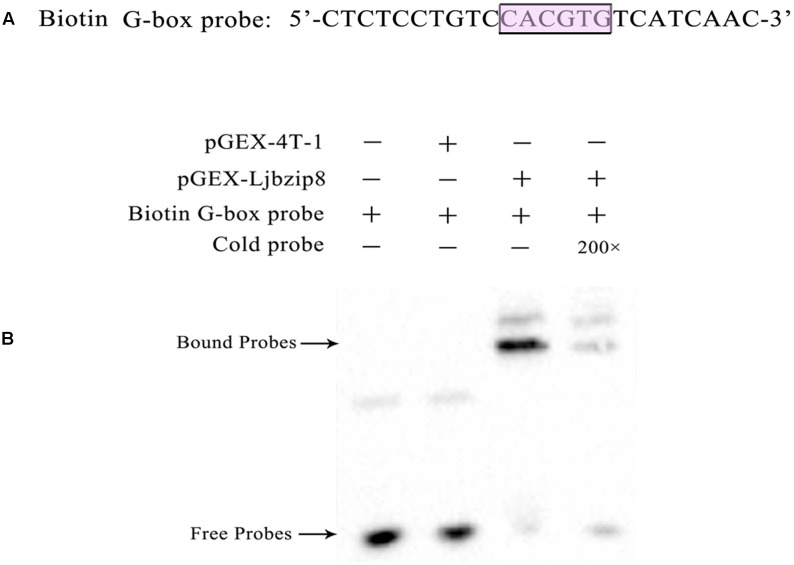
Electrophoretic mobility shift assay with biotin G-box probe and purified recombinant protein *Escherichia coli* BL21 (DE3) [pGEX-LjbZIP8]. **(A)** Sequences of biotin G-box probe. **(B)** EMSA with biotin G-box probe and purified recombinant protein *E. coli* BL21 (DE3)/[pGEX-Ljbzip8]. ‘+’ and ‘-’ indicate the presence and absence of probes, recombinant protein, respectively. “200×” indicate the presence of 200 time concentrations of competitors.

To determine whether LjbZIP and rLjbZIP proteins can bind to the G-box element of the *LjPAL2* Promoter, several bZIP proteins including LjbZIP11 (Group A), rLjbZIP4 (Group A), LjbZIP8 (Group C), rLjbZIP18 (Group C), LjbZIP10 (Group D), and rLjbZIP1 (Group D) were selected for EMSA analysis (Supplementary Table S6). All of the six bZIP proteins were produced in *E. coli* BL21 (DE3) cells, which were subsequently disrupted by sonication (Supplementary Figure S4). The G-box element in the *LjPAL2* 5′-UTR sequence was predicted as CACGTG using Softberry and used as a probe for EMSA analysis (**Figure 2A**).

Electrophoretic mobility shift assay assays showed that purified LjbZIP8 bound specifically to the G-box element and unlabelled probes inhibited the binding (**Figure 2B**). However, rLjbZIP1, rLjbZIP4, rLjbZIP18, LjbZIP10, and LjbZIP18 showed no binding with the G-Box element (Supplementary Figure S5). Further control experiments showed that no binding bands were detected with crude proteins of *E. coli* BL21 (DE3) cells with or without the empty vector. These results clearly demonstrated that LjbZIP8 could bind to the G-box element of the *LjPAL2* 5′-UTR.

### LjbZIP8 Encodes GBF1 Protein Located in the Plant Cell Nucleus

**FIGURE 3:**
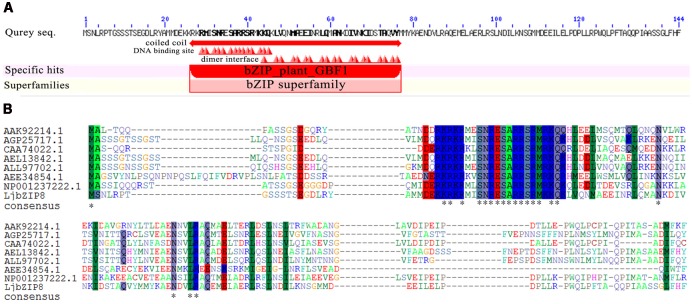
The predicted conserved domains and protein sequence alignment of LjbZIP8. **(A)** The predicted conserved domains of LjbZIP8. **(B)** Protein sequence alignment of LjbZIP8 and other plant bZIP proteins with plant G-box binding 1 factor domain from GenBank. The sequences and their accession numbers are as follows: AAK92214.1 (*Nicotiana tabacum*), AGP25717.1 (*Cicer arietinum*), CAA74022.1 (*Antirrhinum majus*), AEL13842.1 (*Prunus persica*), ALL97702.1 (*Camellia sinensis*), AEE34854.1 (*Arabidopsis thaliana*), and NP_001237222.1 (*Glycine max*).

Bioinformatic analysis revealed that *LjbZIP8* contains a 432-bp ORF that encodes a 144 amino-acid protein. The predicted conserved domains showed that LjbZIP8 belongs to the bZIP GBF1 subfamily with an N-terminal proline-rich domain (**Figure 3A**). Multiple sequence alignments of the deduced LjbZIP8 amino acid sequence revealed that LjbZIP8 has homology with the corresponding enzyme from other plants that has a plant G-box binding 1 factor domain, particularly in the conserved domain at the N-terminal (**Figure 3B**).

**FIGURE 4:**
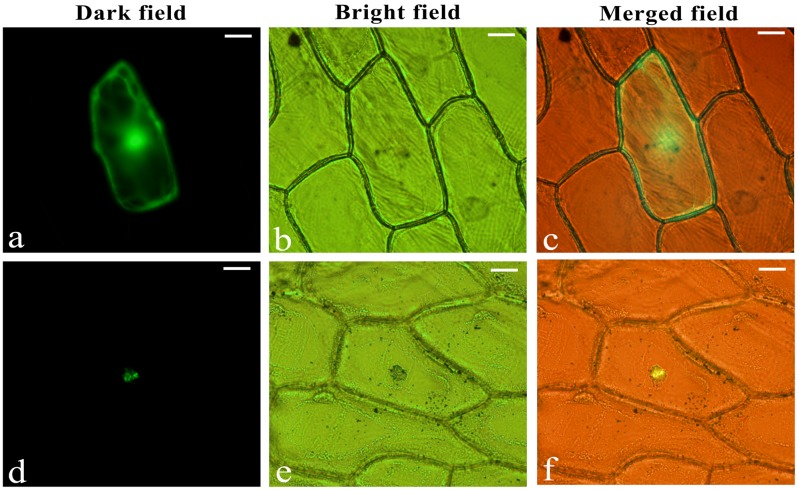
Subcellular localization of LjbZIP8. The recombinant constructs of the LjbZIP8-GFP fusion gene and GFP alone were transformed into onion (*Allium cepa*) epidermal cells by particle bombardment. **(a–c)** Empty vector pE3025; **(d–f)** vector pGEM-LjbZIP8.

To verify the intracellular localization of LjbZIP8, the full-length cDNA sequence of LjbZIP8 was fused in the correct ORF in front of the 5′-terminus of the GFP reporter gene under the control of the CaMV 35S promoter. Onion (*Allium cepa*) epidermal cells were transformed with the *LjbZIP8*-*GFP* construct or a construct containing *GFP* alone by particle bombardment. GFP, without an N-terminal fusion, was localized throughout the cell, while LjbZIP8-GFP was accumulated mainly in the nucleus, suggesting that LjbZIP8 is a nucleus-localized protein (**Figure 4**).

### Transactivation Assay of LjbZIP8

**FIGURE 5:**
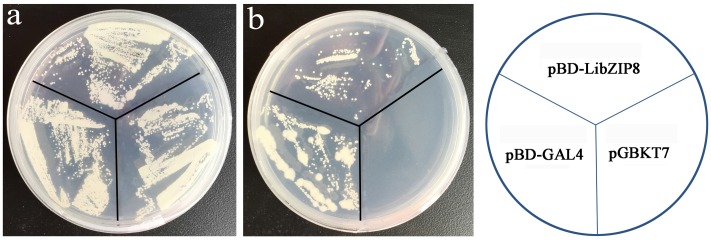
Transactivation assay of LjbZIP8. **(a)** SD-Trp; **(b)** SD-Trp-His. The pBD-GAL4 and pGBKT7 vectors were used as positive and negative controls, respectively.

Most nuclei-localized proteins function as transcription factors. To investigate whether LjbZIP8 had transcriptional activity, we performed transcription activity analysis using the yeast GAL4 system. The full-length cDNA of *LjbZIP8* was fused to the GAL4 DNA binding domain of the pGBKT7 vector to construct pBD-LjbZIP8, which was then used to transform the yeast strain YGR2. Yeasts transformants with pBD-GAL4 and pBD-LjbZIP8 could grow on selection media lacking tryptophan (SD/–Trp) or tryptophan, histidine, and adenine (SD/–Trp/–His/–Ade; **Figure 5**), while those with the empty vector pGBKT7 could not grow on the selection media, suggesting that LjbZIP8 functions as a transcriptional factor.

### Overexpression of LjbZIP8 Changed the CGAs Content in Transgenic Tobacco

To further analyze the role of LjbZIP8 *in vivo*, tobacco was transformed with *LjbZIP8*, and the integration of *LjbZIP8* into the tobacco genome was confirmed using PCR (Supplementary Figure S6). Real-time RT-PCR showed that the expression of *LjbZIP8* was significantly increased in the transgenic plants. Three independent transgenic lines (9, 11, and 17) overexpressing *LjbZIP8* were selected for further analysis. To investigate whether the overexpression of *LjbZIP8* in transgenic tobacco plants affected the accumulation of CGAs, we performed analysis on transgenic leaf samples by HPLC. The content of neochlorogenic acid, CGAs, and cryptochlorogenic acid were lower in transgenic plants overexpressing *LjbZIP8* compared with those in wild-type plants and transgenic plants transformed with the empty vector pCambia1305 (Supplementary Table S7).

**FIGURE 6:**
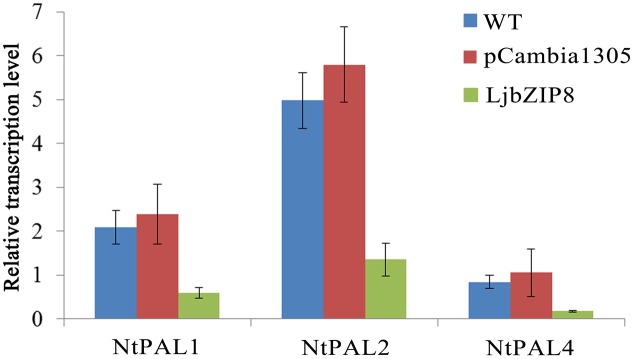
The expression of NtPAL genes in transgenic tobacco. WT, wild-type tobacco; pCambia1305, transgenic tobacco transformed with the empty vector pCambia1305; Ljbzip8, transgenic tobacco overexpressing LjbZIP8.

**FIGURE 7:**
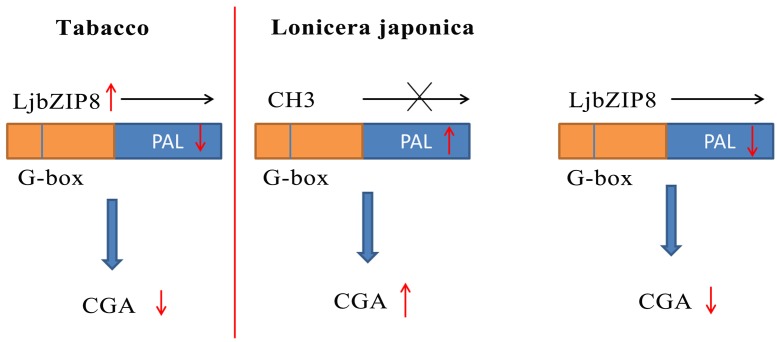
DNA methylation influences CGAs biosynthesis by influencing the recruitment of LjbZIP8.

We further investigated whether the overexpression of *LjbZIP8* affected the expression of *NtPAL*s. The expression levels of *NtPAL1*, *NtPAL2*, and *NtPAL4* were decreased in transgenic tobacco plants compared with wild-type plants (**Figure 6**), indicating that LjbZIP8 might regulate the synthesis of CGAs by inhibiting the expression of *NtPAL*s (**Figure 7**).

### Treatment with 5-Azacytidine Decreases the Transcriptional Level of *LjPAL2* and the Content of CGAs

The DNA methylation inhibitor 5-aza was applied to the leaves of FLJ at a concentration of 80 μM and its effects on the transcriptional level of *LjPAL2* and the content of CGAs were evaluated after 1 day. The results showed that *LjPAL2* expression level of 5-aza-treated leaves was 5.13-fold lower than that in control leaves (Supplementary Figure S7). Furthermore, UPLC analysis showed that the content of neochlorogenic acid, CGAs, cryptochlorogenic acid, isochlorogenic acid A, and isochlorogenic acid C were lower in 5-aza-treated leaves than those in control leaves (**Table 2**), suggesting that lower DNA methylation rates inhibit the expression of *LjPAL2* and accumulation of CGAs in FLJ (**Figure 7**).

**Table 2:** Content of CGAs components in 5-aza treatment of FLJ.

		Content of phenolic components (μg/g)		
No.	Analyte	CK	5-aza-treated	C5-aza/ CCK
1	Neochlorogenic acid	92.41 ± 4.75	28.01 ± 2.24^∗∗∗^	0.30
2	Chlorogenic acid	1944.33 ± 58.55	573.24 ± 18.87^∗∗∗^	0.29
3	Cryptochlorogenic acid	13.61 ± 2.16	7.93 ± 1.97^∗∗^	0.58
4	Isochlorogenic acid B	13.92 ± 7.69	23.69 ± 11.13	1.70
5	Isochlorogenic acid A	129.82 ± 10.43	46.6 ± 2.72^∗∗∗^	0.36
6	Isochlorogenic acid C	13.46 ± 0.21	6.58 ± 1.31^∗∗^	0.49

*The correlation coefficients are marked with ^∗^, ^∗∗^, and ^∗∗∗^ for the significance levels of 0.05, 0.01, and 0.001, respectively*.

## Discussion

Previous research has indicated that several genes, such as *PAL*, *4CL*, *LjC4H*, *LjCCoAOMT1*, *LjF3H*, and *LjGUS*, are differentially expressed between FLJ and rFLJ. However, no differences were observed in the coding region of these genes (unpublished data), suggesting that the differences between FLJ and rFLJ should be attributed to other factors (Yuan et al., 2012). The significant changes in expression of biosynthesis genes may be caused by variations in promoter sequences, such as insertion of transposons (Kobayashi et al., 2004) and other sequences (Espley et al., 2009) into the promoter region, in addition to promoter methylation (Telias et al., 2011). Among these, mC is a fundamental epigenetic mechanism of gene-expression regulation and development in plants. To speculate that increased contents of CGAs in rFLJ may be the result of methylation of the promoters of CGAs biosynthesis genes, the 5′-UTR genomic region of *PAL1*, *PAL2*, *PAL3*, *4CL1*, *4CL2*, *C4H1*, *C4H2*, and *HQT* from FLJ and rFLJ were cloned. Then, the methylation of the 5′-UTR region of these genes were analyzed and methylation of the -109-bp to -279-bp region of the *PAL2* promoter was been found much higher in rFLJ buds than that in FLJ buds. To better understand the differences in DNA methylation between FLJ and rFLJ, we also analyzed methylation of three cytosine types (CHH, CHG, and CG) in the -109-bp to -279-bp regions of *LjPAL2* and found that CG, a cytosine type, had higher methylation levels. Previous investigations have revealed that DNA methylation type that induces color mutation is mainly methylation of CG/CHG cytosine in herbaceous plants (Sekhon and Chopra, 2009). Further, the DNA methylation-based regulatory mechanism is more complicated, since the existence of both CHH and CG/CHG cytosine methylation in the *PcMYB10* promoter increases the stability of the green fruit peril mutant in woody plants (Wang et al., 2013). However, few studies have focused on vine plants, CpG/mCG methylation in FLJ may play a role in the epigenetic regulation of CGAs.

The -109-bp to -279-bp regions, which had higher CpG/mCG methylation levels, may play an important role in the regulation of the *LjPAL2* promoter and, in agreement, we found a G-box-a strong *cis*-acting element in this region. A previous study revealed that G-box sequences were over-represented in eight out of nine promoters of rhythmically co-expressed genes in the flavonol/anthocyanin metabolic pathway, suggesting that HY5 plays an essential role in regulating secondary metabolism in plants through G-box sequences (Pan et al., 2009). OsbZIP71 has been shown to bind to G-box elements specifically, which plays an important role in ABA-mediated drought and salt tolerance in rice (Liu et al., 2014). The bZIP transcription factor HY5 is able to bind to the G-box and promote the transcription of light-induced genes, such as the small subunit of ribulose-1,5-bisphosphate carboxylase/oxygenase (Chattopadhyay et al., 1998). Seventy-five kinds of bZIP factors as a group of transcription factors have been identified in the plant genome, which strictly regulate gene expression in numerous physiological phenomena (Zong et al., 2016). In this study, 11 full-length *LjbZIP* sequences were isolated from the FLJ transcriptome and divided into 10 subgroups based on their similarity to *A. thaliana* bZIP proteins. Our research showed that putative LjbZIP proteins were clustered according to sequence similarities of their basic region. A number of *A. thaliana* bZIP proteins have been predicted to participate in defense against pathogens (Zhou et al., 2000) and stress (Iwata and Koizumi, 2005); however, little is known about whether bZIP proteins control the biosynthesis of CGAs.

5′-azacytidine has effect on too many physiological process, such as improve resistance, anti-oxidation, anti-tumor, induction of flowering, and affect phenotype during the development of plants (Bossdorf et al., 2010; Kondo et al., 2010). General DNA methylation levels of selected genes were decreased with 5-aza-treatment, and the production of resveratrol was increased through a mechanism that involves the induction of STS gene expression in *Vitis amurensis* calli (Kiselev et al., 2011). However, studies have shown that 5-aza could inhibit aflatoxin biosynthesis in *Aspergillus flavus* (Lin et al., 2013). Therefore, it is suggested that 5-aza has different mechanisms for transcriptional regulation of different genes and the accumulation of active compounds. To confirm that DNA methylation affects the expression of *LjPAL2* in FLJ, 5-aza was used to inhibit DNA methylation of FLJ leaves, but *LjPAL2* expression level and the content of CGAs decreased, supporting the negative relationship between DNA methylation and *LjPAL2* expression.

It has been reported that the tobacco bZIP transcription factor BZI-1 binds to G-box elements in the promoters of phenylpropanoid pathway genes *in vitro*; however, BZI-1 could not regulate the biosynthesis of phenylpropanoid (Heinekamp et al., 2002). EMSA analysis also showed that LjbZIP8 could bind to the G-box sequence of the *LjPAL2* 5′-UTR. Conversely, *LjbZIP8* overexpression in transgenic tobacco inhibited the expression of *NtPAL1*, *NtPAL2*, and *NtPAL4*, and decreased the content of neochlorogenic acid, CGAs, and cryptochlorogenic acid, which indicated that *LjbZIP8* could function as a repressor of transcription. Therefore, we speculated that DNA methylation might influence the recruitment of LjbZIP8, regulating *PAL2* expression level and CGAs content in *L. japonica*. It was assumed that variations in cytosine methylation have the potential to play a role in the regulation of active compounds in medicinal plants. However, studies about localization and interaction *in vivo* are required to further understand the mechanism of senescence-specific bZIP regulation.

## Author Contributions

LH and YY conceived and designed the study. LZ and SL performed the experiments and wrote the manuscript. JL, SY, JY, CJ, and YW participated in the research and analyzed the data. Revised the manuscript: LZ and JL. All authors read and approved the final manuscript.

## Conflict of Interest Statement

The authors declare that the research was conducted in the absence of any commercial or financial relationships that could be construed as a potential conflict of interest.

## Footnotes

1 http://bioinfo.ut.ee/primer3/

2 http://genes.mit.edu/GENSCAN.html

3 http://www.psc.edu/biomed/genedoc

4 http://www.arabidopsis.org/

5 http://www.urogene.org/cgi-bin/methprimer/methprimer.cgi

6 http://linux1.softberry.com/berry.phtml

## Abbreviations

ABA: abscisic acid
5-aza: 5-azacytidine
bZIP: basic-leucine zipper
C4H: cinnamate 4-hydroxylase
CGAs: chlorogenic acid
CHS: chalcone synthase
4CL: 4-coumarate CoA ligase
EMSA: electrophoretic mobility shift assay
FLJ: *Lonicera japonica*
GBF: G-box binding factor
HMGR: 3-hydroxy-3-methylglutaryl coenzyme-A reductase
HPLC: high-performance liquid chromatography
HQT: hydroxycinnamoyl-CoA quinate hydroxycinnamoyl transferase
IPTG: isopropyl-β-D-1-thiogalactopyranoside
ORF: open reading frame
PAL: phenylalanine ammonia-lyase
rFLJ: *Lonicera japonica* var. chinensis
SNPs: single nucleotide polymorphisms
UPLC: ultra-performance liquid chromatography
UTR: untranslated regions.

## References

[B1] Altschul S. F. Madden T. L. Schäffer A. A. Zhang J. Zhang Z. Miller W. (1997). Gapped BLAST and PSI-BLAST: a new generation of protein database search programs. *Nucleic Acids Res.* 25 3389–3402. 10.1093/nar/25.17.3389 9254694 PMC146917

[B2] Alves M. S. Dadalto S. P. Gonçalves A. B. De Souza G. B. Barros V. A. Fietto L. G. (2013). Plant bZIP transcription factors responsive to pathogens: a review. *Int. J. Mol. Sci.* 14 7815–7828. 10.3390/ijms14047815 23574941 PMC3645718

[B3] Bossdorf O. Arcuri D. Richards C. L. Pigliucci M. (2010). Experimental alteration of DNA methylation affects the phenotypic plasticity of ecologically relevant traits in *Arabidopsis thaliana*. *Evol. Ecol.* 24 541–553. 10.1007/s10682-010-9372-7

[B4] Burge C. B. Karlin S. (1998). Finding the genes in genomic DNA. *Curr. Opin. Struct. Biol.* 8 346–354. 10.1016/S0959-440X(98)80069-9 9666331

[B5] Chattopadhyay S. Ang L. H. Puente P. Deng X. W. Wei N. (1998). Arabidopsis bZIP protein HY5 directly interacts with light-responsive promoters in mediating light control of gene expression. *Plant Cell* 10 673–683. 10.1105/tpc.10.5.673 9596629 PMC144028

[B6] Chuang C. F. Running M. P. Williams R. W. Meyerowitz E. M. (1999). The PERIANTHIA gene encodes a bZIP protein involved in the determination of floral organ number in *Arabidopsis thaliana*. *Gene. Dev.* 13 334–344. 10.1101/gad.13.3.334 9990857 PMC316427

[B7] Conesa A. Götz S. García-Gómez J. M. Terol J. Talón M. Robles M. (2005). Blast2GO: a universal tool for annotation, visualization and analysis in functional genomics research. *Bioinformatics* 21 3674–3676. 10.1093/bioinformatics/bti610 16081474

[B8] Corrêa L. G. G. Riaño-Pachón D. M. Schrago C. G. dos Santos R. V. Mueller-Roeber B. Vincentz M. (2008). The role of bZIP transcription factors in green plant evolution: adaptive features emerging from four founder genes. *PLoS ONE* 3:e2944 10.1371/journal.pone.0002944 PMC2492810 18698409

[B9] Curradi M. Izzo A. Badaracco G. Landsberger N. (2002). Molecular mechanisms of gene silencing mediated by DNA methylation. *Mol. Cell. Biol.* 22 3157–3173. 10.1128/MCB.22.9.3157-3173 11940673 PMC133775

[B10] Dietrich K. Weltmeier F. Ehlert A. Weiste C. Stahl M. Harter K. (2011). Heterodimers of the *Arabidopsis* transcription factors bZIP1 and bZIP53 reprogram amino acid metabolism during low energy stress. *Plant Cell* 23 381–395. 10.1105/tpc.110.075390 21278122 PMC3051235

[B11] Doyle J. (1991). “DNA protocols for plants,” in *Molecular Techniques in Taxonomy*, eds Hewitt G. M. Johnston A. W. B. Young J. P. W. (Berlin: Springer), 283–293. 10.1007/978-3-642-83962-7_18

[B12] Espley R. V. Brendolise C. Chagné D. Kutty-Amma S. Green S. Volz R. (2009). Multiple repeats of a promoter segment causes transcription factor autoregulation in red apples. *Plant Cell* 21 168–183. 10.1105/tpc.108.059329 19151225 PMC2648084

[B13] Hall T. A. (1999). BioEdit: a user-friendly biological sequence alignment editor and analysis program for Windows 95/98/NT. *Nucl. Acids Symp. Ser.* 41 95–98.

[B14] Heinekamp T. Kuhlmann M. Lenk A. Strathmann A. Dröge-Laser W. (2002). The tobacco bZIP transcription factor BZI-1 binds to G-box elements in the promoters of phenylpropanoid pathway genes in vitro, but it is not involved in their regulation *in vivo*. *Mol. Genet. Genomics* 267 16–26. 10.1007/s00438-001-0636-3 11919711

[B15] Hetzl J. Foerster A. M. Raidl G. Scheid O. M. (2007). CyMATE: a new tool for methylation analysis of plant genomic DNA after bisulphite sequencing. *Plant J.* 51 526–536. 10.1111/j.1365-313X.2007.03152.x 17559516

[B16] Horsch R. B. Fry J. Hoffmann N. Neidermeyer J. Rogers S. G. Fraley R. T. (1989). “Leaf disc transformation,” in *Plant Molecular Biology Manual*, eds Gelvin S. B. Schilperoort R. A. Verma D. P. S. (Dordrecht: Kluwer Academic Publishers), 63–71. 10.1007/978-94-009-0951-9_5

[B17] Iwata Y. Koizumi N. (2005). An Arabidopsis transcription factor, AtbZIP60, regulates the endoplasmic reticulum stress response in a manner unique to plants. *Proc. Natl. Acad. Sci. U.S.A.* 102 5280–5285. 10.1073/pnas.0408941102 15781873 PMC555978

[B18] Jakoby M. Weisshaar B. Dröge-Laser W. Vicente-Carbajosa J. Tiedemann J. Kroj T. (2002). bZIP transcription factors in *Arabidopsis*. *Trends Plant Sci.* 7 106–111. 10.1016/S1360-1385(01)02223-3 11906833

[B19] Jiang X. H. She C. W. Zhu Y. H. Liu X. M. (2014). Cloning and expression analysis of the *Lonicera* japonica Thunb. chlorogenic acid synthetase gene (LjCCoAOMT1) in rice. *Genet. Mol. Res.* 13 2166–2176. 10.4238/2014.March.26.5 24737465

[B20] Jin J. Lian T. Gu C. Yu K. Gao Y. Q. Su X. D. (2016). The effects of cytosine methylation on general transcription factors. *Sci. Rep.* 6:29119 10.1038/srep29119 PMC4935894 27385050

[B21] Jurikova T. Rop O. Mlcek J. Sochor J. Balla S. Szekeres L. (2011). Phenolic profile of edible honeysuckle berries (Genus Lonicera) and their biological effects. *Molecules* 17 61–79. 10.3390/molecules17010061 22269864 PMC6268301

[B22] Kircher S. Ledger S. Hayashi H. Weisshaar B. Schäfer E. Frohnmeyer H. (1998). CPRF4a, a novel plant bZIP protein of the CPRF family: comparative analyses of light-dependent expression, post-transcriptional regulation, nuclear import and heterodimerisation. *Mol. Gen. Genet.* 257 595–605. 10.1007/s004380050687 9604882

[B23] Kiselev K. V. Tyunin A. P. Manyakhin A. Y. Zhuravlev Y. N. (2011). Resveratrol content and expression patterns of stilbene synthase genes in *Vitis amurensis* cells treated with 5-azacytidine. *Plant Cell Tissue Organ. Cult.* 105 65–72. 10.1016/j.plantsci.2010.01.012

[B24] Kobayashi S. Goto-Yamamoto N. Hirochika H. (2004). Retrotransposon -induced mutations in grape skin color. *Science* 304 982–982. 10.1126/science.1095011 15143274

[B25] Kondo H. Shiraya T. Wada K. C. Takeno K. (2010). Induction of flowering by DNA demethylation in *Perilla frutescens* and *Silene armeria*: heritability of 5-azacytidine-induced effects and alteration of the DNA methylation state by photoperiodic conditions. *Plant Sci.* 178 321–326. 10.1016/j.plantsci.2010.01.012

[B26] Kusano T. Berberich T. Harada M. Suzuki N. Sugawara K. (1995). A maize DNA-binding factor with a bZIP motif is induced by low temperature. *Mol. Gen. Genet.* 248 507–517. 10.1007/BF02423445 7476849

[B27] Lee E. J. Kim J. S. Kim H. P. Lee J. H. Kang S. S. (2010). Phenolic constituents from the flower buds of *Lonicera japonica* and their 5-lipoxygenase inhibitory activities. *Food Chem.* 120 134–139. 10.1016/j.foodchem.2009.09.088

[B28] Lescot M. Déhais P. Thijs G. Marchal K. Moreau Y. Van de Peer Y. (2002). PlantCARE, a database of plant cis-acting regulatory elements and a portal to tools for in silico analysis of promoter sequences. *Nucleic Acids Res.* 30 325–327. 10.1093/nar/30.1.325 11752327 PMC99092

[B29] Li L. C. Dahiya R. (2002). MethPrimer: designing primers for methylation PCRs. *Bioinformatics* 18 1427–1431. 10.1093/bioinformatics/18.11.1427 12424112

[B30] Lin H. B. Peng Y. D. Guan R. W. Lu J. X. Wang M. Liu G. X. (2015). Comparative study of the content of chlorogenic acid in different Germplasms of *Lonicera japonica* flos. *Lishizhen Med. Mater. Med. Res.* 26 1755–1756. 10.1016/j.plaphy.2016.12.027

[B31] Lin J. Q. Zhao X. X. Wang C. C. Xie Y. Li G. H. He Z. M. (2013). 5-azacytidine inhibits aflatoxin biosynthesis in *Aspergillus flavus*. *Ann. Microbiol.* 63 763–769. 10.1007/s13213-012-0531-7

[B32] Liu C. Mao B. Ou S. Wang W. Liu L. Wu Y. (2014). OsbZIP71, a bZIP transcription factor, confers salinity and drought tolerance in rice. *Plant Mol. Biol.* 84 19–36. 10.1007/s11103-013-0115-3 23918260

[B33] Lozano-Sotomayor P. Chávez Montes R. A. Silvestre-Vañó M. Herrera- Ubaldo H. Greco R. Pablo-Villa J. (2016). Altered expression of the bZIP transcription factor DRINK ME affects growth and reproductive development in *Arabidopsis thaliana*. *Plant J.* 88 437–451. 10.1111/tpj.13264 27402171

[B34] Ordway J. M. Bedell J. A. Citek R. W. Nunberg A. Garrido A. Kendall R. (2006). Comprehensive DNA methylation profiling in a human cancer genome identifies novel epigenetic targets. *Carcinogenesis* 27 2409–2423. 10.1093/carcin/bgl161 16952911

[B35] Pan Y. Michael T. P. Hudson M. E. Kay S. A. Chory J. Schuler M. A. (2009). Cytochrome P450 monooxygenases as reporters for circadian -regulated pathways. *Plant Physiol.* 150 858–878. 10.1104/pp.108.130757 19386812 PMC2689971

[B36] Pattengale N. D. Alipour M. Bininda-Emonds O. R. Moret B. M. Stamatakis A. (2010). How many bootstrap replicates are necessary? *J. Comput. Biol.* 17 337–354. 10.1089/cmb.2009.0179 20377449

[B37] Peredo E. L. Revilla M. Á and Arroyo-Garc ía R. (2006). Assessment of genetic and epigenetic variation in hop plants regenerated from sequential subcultures of organogenic calli. *J. Plant Physiol.* 163 1071–1079. 10.1016/j.jplph.2005.09.010 16971217

[B38] Pharmacopoeia of the People’s Republic of China. (2015). *The Pharmacopoeia Committee of China.* Beijing: Chemical Industry Press.

[B39] Sekhon R. S. Chopra S. (2009). Progressive loss of DNA methylation releases epigenetic gene silencing from a tandemly repeated maize Myb gene. *Genetics* 181 81–91. 10.1534/genetics.108.097170 19001287 PMC2621191

[B40] Seo O. N. Kim G. S. Park S. Lee J. H. Kim Y. H. Lee W. S. (2012). Determination of polyphenol components of Lonicera japonica Thunb. using liquid chromatography–tandem mass spectrometry: contribution to the overall antioxidant activity. *Food Chem.* 134 572–577. 10.1016/j.foodchem.2012.02.124 24176305

[B41] Shan J. H. Fu J. Zha Z. H. Kong X. Q. Huang H. Luo L. (2009). Chlorogenic acid inhibits lipopolysaccharide-induced cyclooxygenase-2 expression in RAW264. 7 cells through suppressing NF-κB and JNK/AP-1 activation. *Int. Immunopharmacol.* 9 1042–1048. 10.1016/j.intimp.2009.04.011 19393773

[B42] Shang X. Pan H. Li M. X. Miao X. L. Ding H. (2011). *Lonicera japonica* Thunb.: ethnopharmacology, phytochemistry and pharmacology of an important traditional Chinese medicine. *J. Ethnopharmacol.* 138 1–21. 10.1016/j.jep.2011.08.016 21864666 PMC7127058

[B43] Shinozuka H. Cogan N. O. Shinozuka M. Marshall A. Kay P. Lin Y. H. (2015). A simple method for semi-random DNA amplicon fragmentation using the methylation-dependent restriction enzyme MspJI. *BMC Biotechnol.* 15:25 10.1186/s12896-015-0139-7 PMC4396059 25887558

[B44] Sibéril Y. Benhamron S. Memelink J. Giglioli-Guivarc’h N. Thiersault M. Boisson B. (2001). *Catharanthus roseus* G-box binding factors 1 and 2 act as repressors of strictosidine synthase gene expression in cell cultures. *Plant Mol. Biol.* 45 477–488. 10.1023/A:1010650906695 11352466

[B45] Strathmann A. Kuhlmann M. Heinekamp T. Dröge-Laser W. (2001). BZI-1 specifically heterodimerises with the tobacco bZIP transcription factors BZI-2 BZI-3/TBZF and BZI-4 and is functionally involved in flower development. *Plant J.* 28 397–408. 10.1046/j.1365-313X.2001.01164.x 11737777

[B46] Tamura K. Stecher G. Peterson D. Filipski A. Kumar S. (2013). MEGA6: molecular evolutionary genetics analysis version 6.0. *Mol. Biol. Evol.* 30 2725–2729. 10.1093/molbev/mst197 24132122 PMC3840312

[B47] Tatusov R. L. Koonin E. V. Lipman D. J. (1997). A genomic perspective on protein families. *Science* 278 631–637. 10.1126/science.278.5338.631 9381173

[B48] Telias A. Lin-Wang K. Stevenson D. E. Cooney J. M. Hellens R. P. Allan A. C. (2011). Apple skin patterning is associated with differential expression of MYB10. *BMC Plant Biol.* 11:93 10.1186/1471-2229-11-93 PMC3127826 21599973

[B49] Thompson J. D. Gibson T. Higgins D. G. (2002). Multiple sequence alignment using ClustalW and ClustalX. *Curr. Protoc. Bioinformatics* Chapter 2:Unit 2.3. 10.1002/0471250953.bi0203s00 18792934

[B50] Wang Z. Meng D. Wang A. Li T. Jiang S. Cong P. (2013). The methylation of the PcMYB10 promoter is associated with green-skinned sport in Max Red Bartlett pear. *Plant Physiol.* 162 885–896. 10.1104/pp.113.214700 23629835 PMC3668077

[B51] Weltmeier F. Rahmani F. Ehlert A. Dietrich K. Schütze K. Wang X. (2009). Expression patterns within the *Arabidopsis* C/S1 bZIP transcription factor network: availability of heterodimerization partners controls gene expression during stress response and development. *Plant Mol. Biol.* 69 107–119. 10.1007/s11103-008-9410-9 18841482 PMC2709229

[B52] Xiang C. B. Miao Z. H. Lam M. E. (1997). DNA-binding properties, genomic organization and expression pattern of TGA6 a new member of the TGA family of bZIP transcription factors in *Arabidopsis thaliana*. *Plant Mol. Biol.* 34 403–415. 10.1023/A:1005873500238 9225852

[B53] Yuan Y. Song L. Li M. Liu G. Chu Y. Ma L. (2012). Genetic variation and metabolic pathway intricacy govern the active compound content and quality of the Chinese medicinal plant *Lonicera japonica* thunb. *BMC Genomics* 13:195 10.1186/1471-2164-13-195 PMC3443457 22607188

[B54] Yuan Y. Wang Z. Y. Jiang C. Wang X. M. Huang L. Q. (2014). Exploiting genes and functional diversity of chlorogenic acid and luteolin biosyntheses in *Lonicera japonica* and their substitutes. *Gene* 534 408–416. 10.1016/j.gene.2012.09.051 23085319 PMC7138419

[B55] Yuan Y. Wu C. Liu Y. J. Yang J. Huang L. Q. (2013). The *Scutellaria baicalensis* R2R3-MYB transcription factors modulates flavonoid biosynthesis by regulating GA metabolism in transgenic tobacco plants. *PLoS ONE* 8:e77275 10.1371/journal.pone.0077275 PMC3797077 24143216

[B56] Zdobnov E. M. Apweiler R. (2001). InterProScan–an integration platform for the signature-recognition methods in InterPro. *Bioinformatics* 17 847–848. 10.1093/bioinformatics/17.9.847 11590104

[B57] Zha L. Liu S. Su P. Yuan Y. Huang L. (2016). Cloning, prokaryotic expression and functional analysis of squalene synthase (SQS) in *Magnolia officinalis*. *Protein Expr. Purif.* 120 28–34. 10.1016/j.pep.2015.12.008 26696600

[B58] Zhou J. M. Trifa Y. Silva H. Pontier D. Lam E. Shah J. (2000). NPR1 differentially interacts with members of the TGA/OBF family of transcription factors that bind an element of the PR-1 gene required for induction by salicylic acid. *Mol. Plant Microbe Interact.* 13 191–202. 10.1094/MPMI.2000.13.2.191 10659709

[B59] Zong W. Tang N. Yang J. Peng L. Ma S. Xu Y. (2016). Feedback regulation of ABA signaling and biosynthesis by a bZIP transcription factor targets drought resistance related genes. *Plant Physiol.* 171 2810–2825. 10.1104/pp.16.00469 27325665 PMC4972276

