# Supplementary material for: DNA Methylation Influences Chlorogenic Acid Biosynthesis in Lonicera japonica by Mediating LjbZIP8 to Regulate Phenylalanine Ammonia-Lyase 2 Expression
Source: Front Plant Sci. 2017 Jul 10;8:1178. doi: 10.3389/fpls.2017.01178 (PMC5502268; doi:10.3389/fpls.2017.01178)
Supplement: Supplementary file 1 [file Data_Sheet_1.DOCX]

**Supplemental materials**


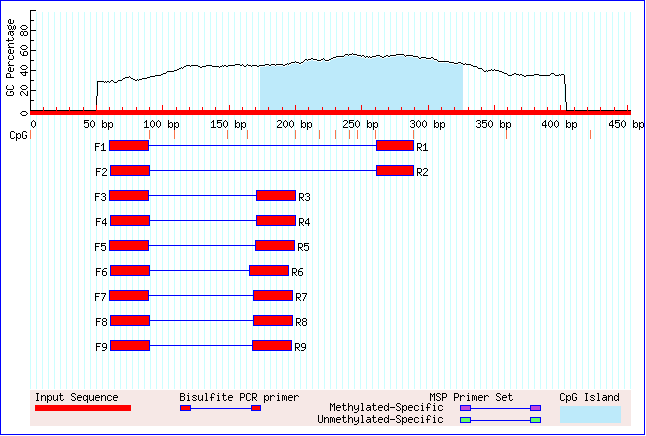


**Supplementary Figure S1.** CpG island prediction results of LjPAL2


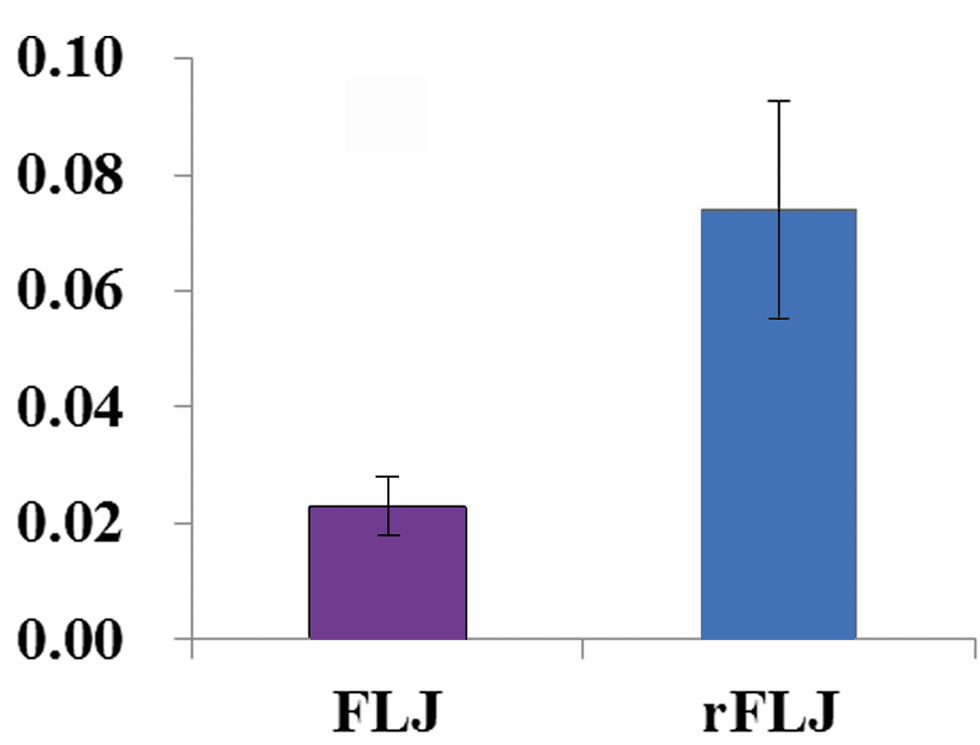


**Relative transcription level**

**Supplementary Figure S2.** The relative transcription level of *LjPAL2* Between buds of *L. japonica* (FLJ) and *L. japonica* Thunb. var. chinensis (rFLJ).


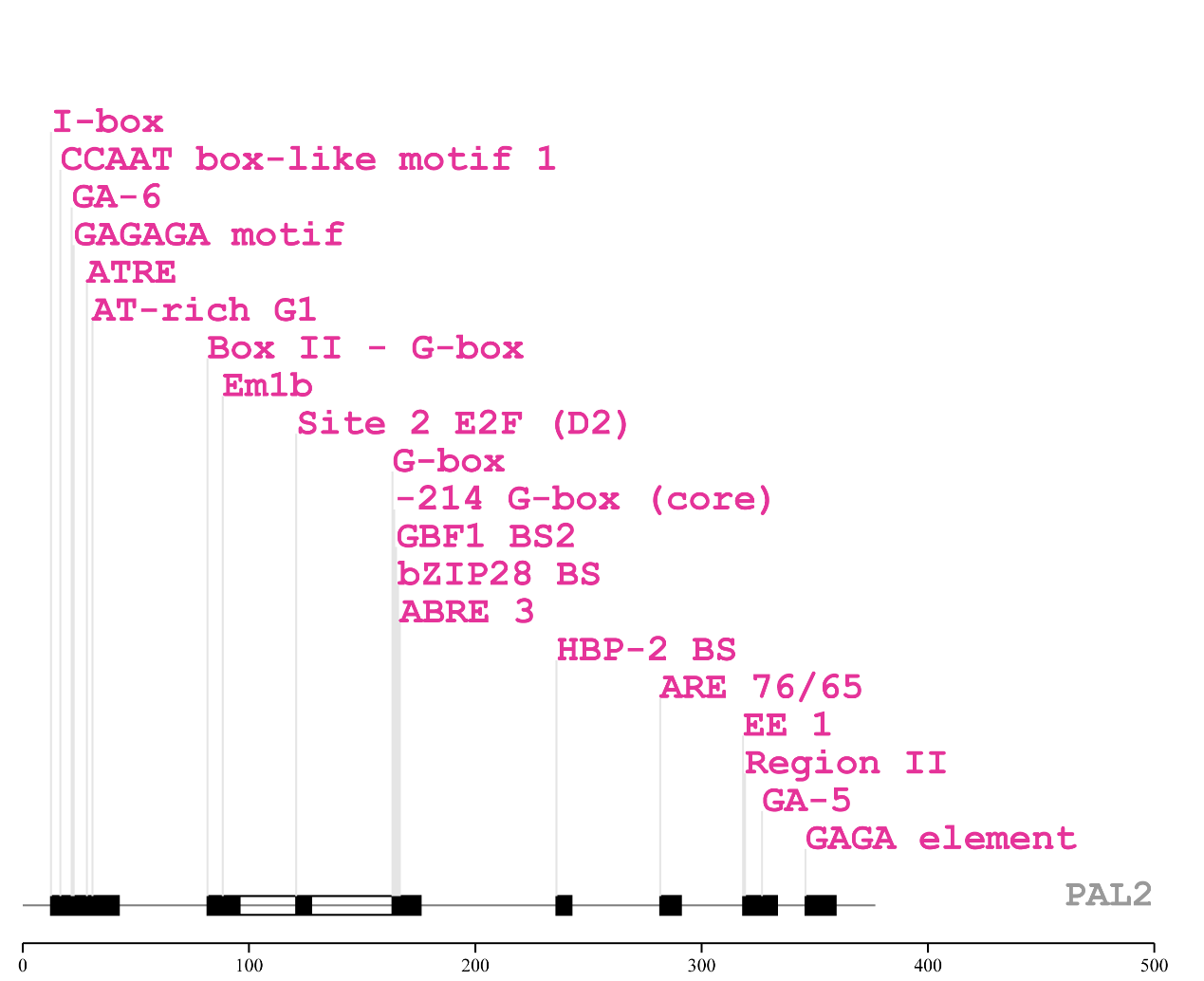


**Supplementary Figure S3.** Regulatory elements in the CpG loci in 5’-flanking region of LjPAL2.

**
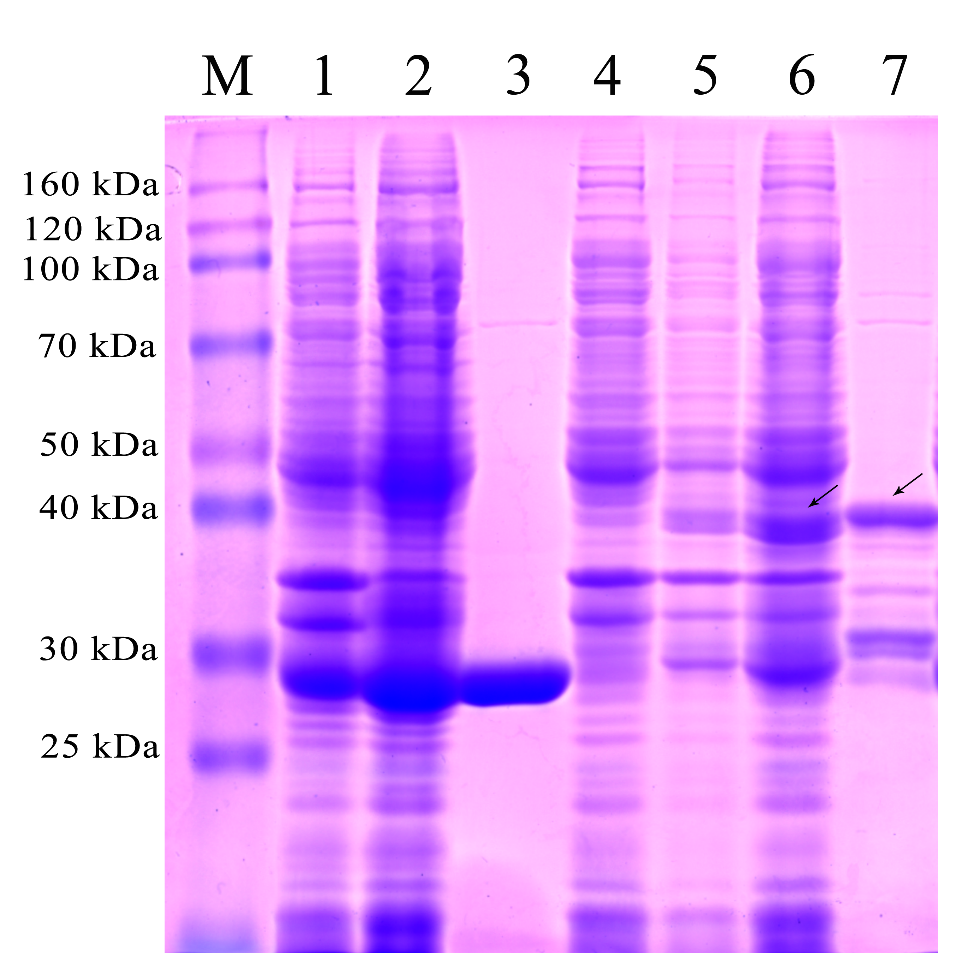
**

**Supplementary Figure S4**. Expression of LjbZIP8 protein in *E. coli* BL21(DE3).

**M:** protein molecular weight standards; **1:** *E.coli* BL21(DE3)/pGEX-4T-1 total cell extract induced with IPTG at 16 °C; **2:** *E.coli* BL21(DE3)/pGEX-4T-1 cell precipitate induced with IPTG at 16 °C; **3:** *E.coli* BL21(DE3)/pGEX-4T-1 purified protein induced with IPTG at 16 °C; **4:** *E. coli* BL21( DE3) /［pGEX-LjbZIP8］total cell extract induced with IPTG at 16 °C; **5:** *E. coli* BL21( DE3) /［pGEX-LjbZIP8］total cell extract without IPTG;6*:E. coli* BL21( DE3) /［pGEX-LjbZIP8］cell precipitate induced with IPTG at 16 °C; **7:** *E.coli* BL21(DE3)/［pGEX-LjbZIP8］purified protein induced with IPTG at 16 °C;





**Supplementary Figure S5.** EMSA with biotin G-box probe and purified recombinant protein *E.coli* BL21(DE3) [pGEX-LjbZIPs].

A,D,G: *E. coli* BL21(DE3) [pGEX-4t-1]; B,C: *E. coli* BL21(DE3) [pGEX-rLJbzip4]; E,F: *E. coli* BL21(DE3) [pGEX- LjbZIP10]; G,H: *E. coli* BL21(DE3) [pGEX- rLJbzip]; B,E,H: biotin labeled G-box probe; C,F, I:biotin labeled and unlabeled G-box probes; J: no proteins added in blank control group.


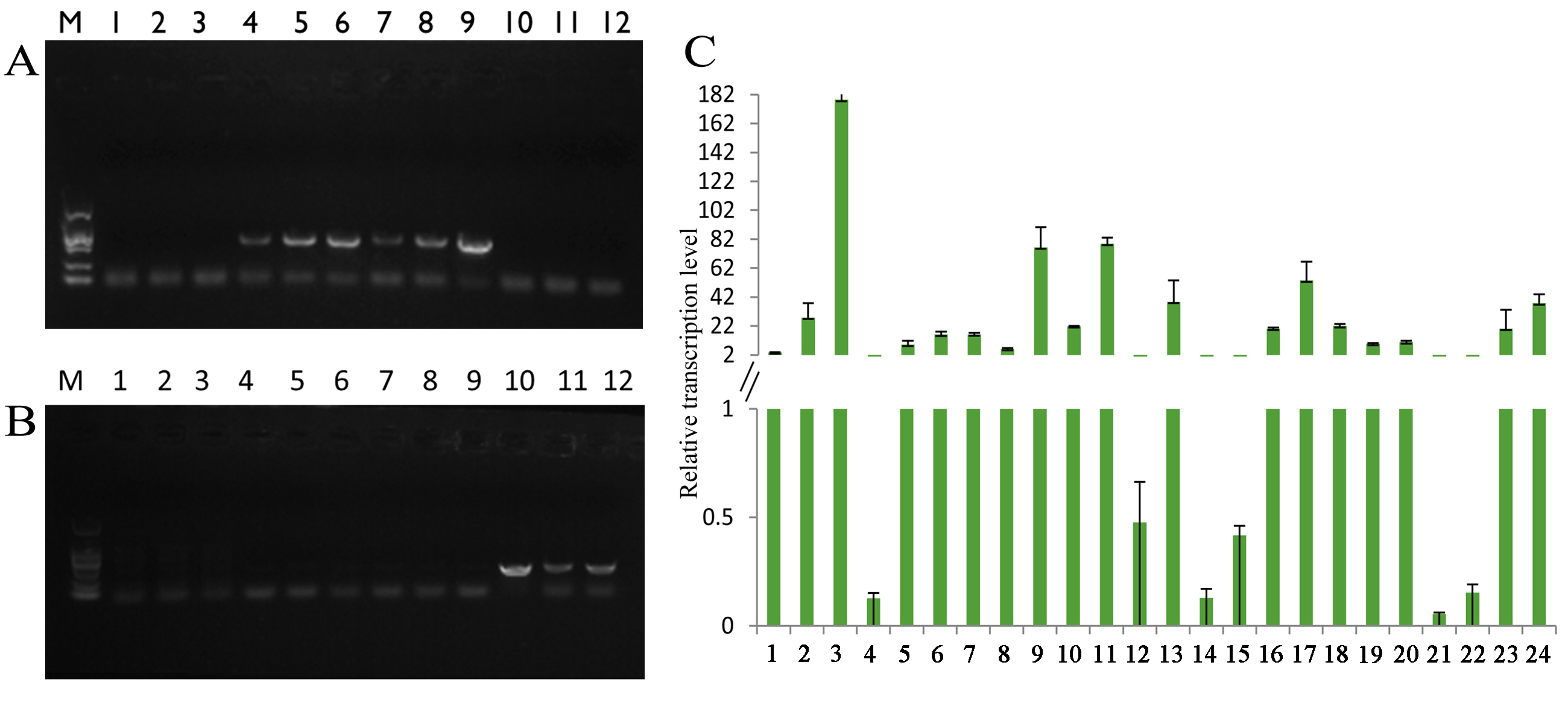


**A**

**Supplementary Figure S6.** PCR analysis of transgenic tobacco.

**(A)** hpt gene, M, 2000bp DNA ladder; 1-3, wild-type tobacco; 4-6, p1305; 7-9, transgenic tobacco; 10-12, H_2_O; **(B)** Ljbzip8 gene, M, 2000bp DNA ladder; 1-3, wild-type tobacco; 4-6, p1305; 7-9, H_2_O; 10-12, transgenic tobacco; **(C)** Real-time PCR analysis of *Ljbzip8* in transgenic tobacco plants. Transgenic tobacco plants with high transcription level of *Ljbzip8* were chosen for the next transgenic experiments.


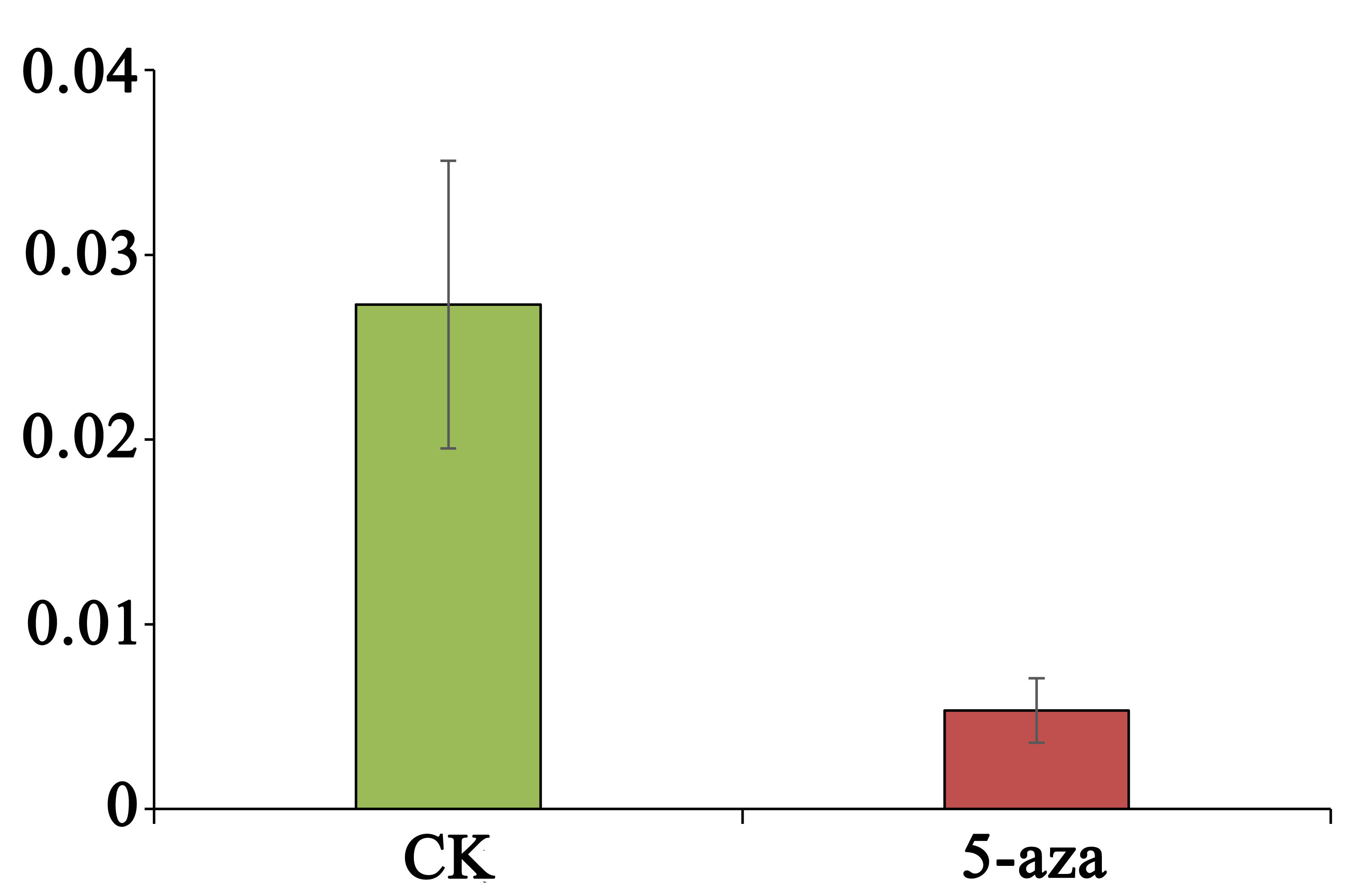


**Relative transcription level**

**Supplementary Figure S7.** **The transcription level of LjPAL2 between leaves of 5’azacytidine treated *L. japonica* (5-aza) and wild-type *L. japonica* (CK).**


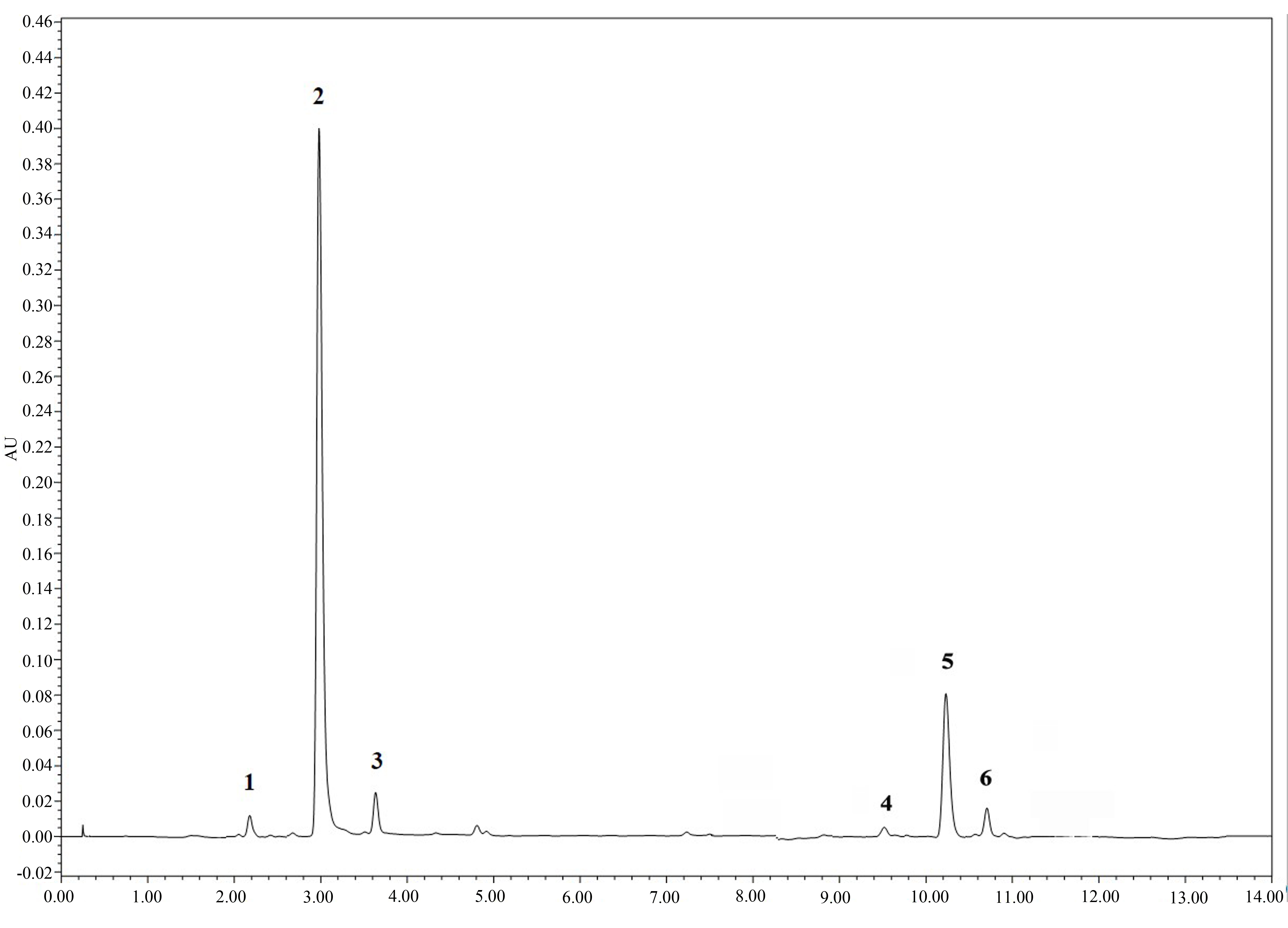


**Supplementary Figure S8.** UPLC chromatograms at 340 nm of CGAs constituents from *L. japonica*.

1. neochlorogenic acid; 2. chlorogenic acid; 3. cryptochlorogenic acid;

4. isochlorogenic acid B; 5. isochlorogenic acid A; 6. isochlorogenic acid C .

**Supplemental Table S1. Primers used in this study**

| **Primers** | **Sequences (5’ to 3 ’)** |  |
| --- | --- | --- |
| **Bisulfite Sequencing** | |  |
| Methy-PAL1.F | TAAATAATAATTTTTTATTTAGTATTTAAA |  |
| Methy-PAL1.R | AAATTCTTTTTCACTTATCAAAAAC |  |
| Methy-PAL2.F | TTTAGATAAATAGAGAGTTAAGAGATATAT |  |
| Methy-PAL2.R | TTAAATAAAAAATAAATTAACTAAAAAC |  |
| Methy-PAL3.F | TAAATTTATTGTATATAATTTTTTTGTTTT |  |
| Methy-PAL3.R | TATATTTTCCTAAAATTTCTCTAAAAATTA |  |
| Methy-4CL1.F | TTTTGGTAGTAGGTAAAAAGTGTTTT |  |
| Methy-4CL1.R | AAAAATAAATCCAAAATTAAATAAC |  |
| Methy-4CL2.F | TTTTTTAATATTTAAATTTAGATAATTGTA |  |
| Methy-4CL2.R | TTCTAATAAACTTCAAAATAAATCCATATC |  |
| Methy-CHI2.F | TTTAAATAAAGGAATTTGGTTAAAAA |  |
| Methy-CHI2.R | AAACCAACTTAACCCAAAAAATAAC |  |
| Methy-CHS2.F | TGTATAAGAATTATTGTTTGGAGTAGTTTA |  |
| Methy-CHS2.R | AAAAAAAATATTTAATAACAAAAAAAA |  |
|  |  |  |
| **Real-time PCR in *L. japonica* and *L. japonica* Thunb. var. chinensis** | |  |
| LjbZIP1.F | CTACGACGGGTATTGGC |  |
| LjbZIP1.R | TTGGCTTTGATTCTTCTTC |  |
| LjbZIP2.F | CTTTAACCCTCACCTCC |  |
| LjbZIP2.R | ATCAGAAGAGTGCCAGAG |  |
| LjbZIP3.F | CAAGTAGAACTGGAGCAA |  |
| LjbZIP3.R | AACGATGACAGAGGAGC |  |
| LjbZIP4.F | GGTCACAACGAGCCTAA |  |
| LjbZIP4.R | CCTCCCTCTGGTTTCAT |  |
| LjbZIP5.F | TGATGGAAAAAGTGGAGAAAGT |  |
| LjbZIP5.R | CCCGCAACCAGGAAGGT |  |
| LjbZIP6.F | ACAGAACTAGATCCCAATG |  |
| LjbZIP6.R | ACTCCAACCCGATACAA |  |
| LjbZIP7.F | TTGTCGCCCTCTACTTC |  |
| LjbZIP7.R | TTCTCCTTAGCCTCCTC |  |
| LjbZIP8.F | CATTATCCGATACACCAAAGCCG |  |
| LjbZIP8.R | CGAACGTGCAGCAGACTCCC |  |
| LjbZIP9.F | CTGTTTTGAGGGCTCAGATGG |  |
| LjbZIP9.R | TTCTGTTATATCATAGATTTCATTGT |  |
| LjbZIP10.F | AGACAATCTACGGCAGCAAACA |  |
| LjbZIP10.R | CAAGCCAAAGAGAGCTAAGGG |  |
| LjbZIP11.F | CGATGCCCCACATAATAGACC |  |
| LjbZIP11.R | GAACCGATTGCCCCTGC |  |
| LjPAL2.F | TGCCGAAAGAAGTCGAAAGT |  |
| LjPAL2.R | TGTCAAATACTCGCCCTTCA |  |
| **Expression of LjbZIP proteins in *E.coli*** | |  |
| LjbZIP11-BamHI. F | GGATCCGATGGGGATTCAGACAATGGGAT |  |
| LjbZIP11-SalI.R | GTCGACTTATAGCGAACTTGTTCTTCGAAGC |  |
| LjbZIP8-BamHI.F | CCCGGGGATGGCAAATTCCAAGGGGC |  |
| LjbZIP8-SalI.R | GCGGCCGCTCAGATGTGGAGCGGGCCGGTAAC |  |
| LjbZIP10-BamHI.F | GGATCCGATGTCGAATTTGAGGCCAACTG |  |
| LjbZIP10-SalI.R | GTCGACTTAGAAATGAAACAACCCGGAAG |  |
| **Subcellular localization** |  | |
| E3025-LjbZIP8-.F | CCATGGATGTCGAAATTTGAG | |
| E3025-LjbZIP8-.R | GGTACCTTAGAAATGAAACAACC | |
| **Transactivation assay** |  | |
| LjbZIP8-EcoRI.F | GAATTCATGTCGAATTTGAGGCCA | |
| LjbZIP8-SalI.R | GTCGACTTAGAAATGAAACAACCCG | |
| **Plant expression vector** |  | |
| 182084-spel.r | ACTAGTATGTCGAATTTGAGGC | |
| 182084-pmli.f | CACGTGTTAGAAATGAAACAAC | |
| **PCR detection in transgenic tobacco** | | |
| LjbZIP8-F | ATGTCGAATTTGAGGCCAACTG | |
| LjbZIP8-R | TTAGAAATGAAACAACCCGGAAG | |
| HYG-F | AGCTGCGCCGATGGTTTCTAC | |
| HYG-R | ATCGCCTCGCTCCAGTCAATG | |
| **Real-time PCR in transgenic tobacco** | | |
| NtACT.qF | TCCTGATGGGCAAGTGATTAC | |
| NtACT.qR | TTGTATGTGGTCTCGTGGATTC | |
| NtPAL1.qF | ACAACACAACCAAGATGTCAACTCC | |
| NtPAL1.qR | TCTTTAAGTTTTCTTCCAAATGCC | |
| NtPAL2.qF | CCAGGATCTTTCTACCCATCC | |
| NtPAL2.qR | GTGCAACACCAGCCATTTTT | |
| NtPAL4.qF | CAACTAAGATGATTGAGAGGGAGAT | |
| NtPAL4.qR | TGGACACACCGATAGGGG | |
| LjbZIP8.qF | GAGGGCTCAAGAAATGGAAT | |
| LjbZIP8.qR | GCTGCCACGGTCTCAACAG | |
| **Quantitative DNA methylation analysis** | | |
| PAL2-Pro.F2 | ACACCACGCATACCTCATTTCA | |
| PAL2-Pro.R2 | CCATTGCTATTGCCATTCTCC | |

**Supplemental Table S2.** Putative CpG methylation in 5’UTR region of genes involved in the CGAs and luteolin biosynthesis

| **Gene Name** | **GenBank No** | **5’UTR length(bp)** | **CpG methylation** | **CpGs in product** |
| --- | --- | --- | --- | --- |
| *LjPAL1* | JX068601 | 1097 | NO |  |
| *LjPAL2* | JX068602 | 452 | Yes | 24 |
| *LjPAL3* | JX068603 | 2039 | NO |  |
| *Lj4CL1* | JX068604 | 1626 | NO |  |
| *Lj4CL2* | JX068605 | 514 | NO |  |
| *LjC4H1* | JX068606 | 657 | Yes | 20 |
| *LjC4H2* | JX068607 | 207 | NO |  |
| *LjCHS1* | JX068608 | 489 | NO |  |
| *LjCHS2* | JX068609 | 684 | NO |  |
| *LjCHI1* | JX068610 | 135 | NO |  |
| *LjCHI2* | JX068611 | 2796 | NO |  |
| *LjHQT* | ACZ52698 | 456 | NO |  |
| *LjFNS* | JX068612 | 1065 | NO |  |

**Supplemental Table S3.** DNA methylation ratio of *PAL2* promoter in *L. japonica* and *L. japonica* var.chinensis

|  | **Species** | **N0.** | **DNA methylation ratio of *PAL2* promoter** |
| --- | --- | --- | --- |
| Population A | *L. japonica* var.chinensis | A-1 | 0.9406 |
|  | *L. japonica* var.chinensis | A-2 | 0.9658 |
|  | *L. japonica* var.chinensis | A-3 | 0.9441 |
|  | *L. japonica* var.chinensis | A-4 | 0.8800 |
|  | *L. japonica* var.chinensis | A-5 | 0.9676 |
|  | *L. japonica* var.chinensis | A-6 | 0.9337 |
|  | *L. japonica* var.chinensis | A-7 | 0.8893 |
|  | *L. japonica* var.chinensis | A-8 | 0.6982 |
|  | *L. japonica* var.chinensis | A-9 | 0.8127 |
|  | *L. japonica* var.chinensis | A-10 | 0.7667 |
| Population B | *L. japonica* var.chinensis | B-1 | 0.7297 |
|  | *L. japonica* var.chinensis | B-2 | 0.9090 |
|  | *L. japonica* var.chinensis | B-3 | 0.8913 |
|  | *L. japonica* var.chinensis | B-4 | 0.8890 |
|  | *L. japonica* var.chinensis | B-5 | 0.7402 |
|  | *L. japonica* var.chinensis | B-6 | 0.6919 |
|  | *L. japonica* var.chinensis | B-7 | 0.8662 |
|  | *L. japonica* var.chinensis | B-8 | 0.9366 |
|  | *L. japonica* var.chinensis | B-9 | 0.8383 |
| Population C | *L. japonica* var.chinensis | C-1 | 0.7868 |
|  | *L. japonica* var.chinensis | C-2 | 0.9441 |
|  | *L. japonica* var.chinensis | C-3 | 0.7659 |
|  | *L. japonica* var.chinensis | C-4 | 0.6968 |
|  | *L. japonica* var.chinensis | C-5 | 0.6926 |
|  | *L. japonica* var.chinensis | C-6 | 0.7668 |
|  | *L. japonica* var.chinensis | C-7 | 0.8762 |
|  | *L. japonica* var.chinensis | C-8 | 0.4046 |
|  | *L. japonica* var.chinensis | C-9 | 0.3946 |
|  | *L. japonica* var.chinensis | C-10 | 0.7105 |
| Population A | *L.japonica* | A-1 | 0.6037 |
|  | *L.japonica* | A-2 | 0.2432 |
|  | *L.japonica* | A-3 | 0.0215 |
|  | *L.japonica* | A-4 | 0.3458 |
|  | *L.japonica* | A-5 | 0.0390 |
|  | *L.japonica* | A-6 | 0.0131 |
|  | *L.japonica* | A-7 | 0.4634 |
| Population B | *L.japonica* | B-1 | 0.4117 |
|  | *L.japonica* | B-2 | 0.7949 |
|  | *L.japonica* | B-3 | 0.8163 |
|  | *L.japonica* | B-4 | 0.8258 |
|  | *L.japonica* | B-5 | 0.7851 |
|  | *L.japonica* | B-6 | 0.8899 |
|  | *L.japonica* | B-7 | 0.8385 |
|  | *L.japonica* | B-8 | 0.8256 |
|  | *L.japonica* | B-9 | 0.0981 |
|  | *L.japonica* | B-10 | 0.5550 |
| Population C | *L.japonica* | C-1 | 0.7735 |
|  | *L.japonica* | C-2 | 0.7542 |
|  | *L.japonica* | C-3 | 0.7510 |
|  | *L.japonica* | C-4 | 0.9102 |
|  | *L.japonica* | C-5 | 0.8941 |
|  | *L.japonica* | C-6 | 0.6644 |
|  | *L.japonica* | C-7 | 0.9255 |
|  | *L.japonica* | C-8 | 0.1630 |
|  | *L.japonica* | C-9 | 0.6813 |
|  | *L.japonica* | C-10 | 0.5740 |
| Population D | *L.japonica* | D-1 | 0.8781 |
|  | *L.japonica* | D-2 | 0.7386 |
|  | *L.japonica* | D-3 | 0.7740 |
|  | *L.japonica* | D-4 | 0.7925 |
|  | *L.japonica* | D-5 | 0.7836 |
|  | *L.japonica* | D-6 | 0.7854 |
|  | *L.japonica* | D-7 | 0.7627 |
|  | *L.japonica* | D-8 | 0.7631 |
|  | *L.japonica* | D-9 | 0.6516 |
|  | *L.japonica* | D-10 | 0.0169 |

**Supplementary Table S4.** Putative transcription factor binding sites and regulatory elements in the upstream region of *LjPAL2*

| **Gene** | **RE** | **BF** |
| --- | --- | --- |
| Synthetic oligonucleotides | G-box | Atbzip1 |
| RBCS-1A | G box-1 | HY5 |
| AtEm6/1 | ABRE/6.2/1.2 | ABI5 |
| Adh | -214G-box (core) | GBF3 |
| RbcS-1A | G box-2 | GBF |
| RD29B | ABRE 3 | ABI3; ABI5; AREB1 |
| Synthetic oligonucleotides | GBF1 BS2 | GBF1 |
| ARF18 (At1g23750) | G-box | STF1/HY5 |
| IAA17/AXR3 (At1g04250) | G-box | STF1/HY5 |
| Synthetic oligonucleotides | G-box | AtMYC2/JIN1 (ZBF1) |
| Synthetic oligonucleotides | bZIP28 BS | bZIP28 |
| PSY | G-box | PIF1 |

**Supplementary Table S5.** Overall analysis of bZIP genes in *L. japonica*

| **Gene** | **GenBank No** | **Putative function** | **Species** | **GenBank No.** | **E value** | **qPCR** |
| --- | --- | --- | --- | --- | --- | --- |
| LjbZIP1 | KT218625 | bZIP transcription factor family protein | *Populus trichocarpa* | XP_006381298.1 | 3e-47 | 1.0015 |
| LjbZIP2 | KT218626 | transcription factor HY5 | *Betula platyphylla* | AHY20043.1 | 8e-61 | 0.9990 |
| LjbZIP3 | KT218627 | basic region leucine zipper protein | Nicotiana tabacum | BAF76429.1 | 3e-50 | 0.9970 |
| LjbZIP4 | KT218628 | bZIP transcription factor family protein 2 | Camellia sinensis | AFP19453.1 | 8e-67 | 0.9789 |
| LjbZIP5 | KT218629 | transcriptional activator TAF-1-like | Solanum tuberosum | XP_006339668.1 | 3e-160 | 0.9980 |
| LjbZIP6 | KT218630 | basic leucine zipper 6-like isoform X1 | Populus euphratica | XP_011024148.1 | 4e-60 | 1.0005 |
| LjbZIP7 | KT218631 | bZIP transcription factor family protein 3 | Camellia sinensis | AGD98701.1 | 1e-112 | 1.0008 |
| LjbZIP8 | KT218632 | bZIP G-box binding factor1 (GBF1) subfamily | Solanum lycopersicum | NP_001234505.1 | 4e-43 | 0.9987 |
| LjbZIP9 | KT218633 | putative transcription factor kapC | Sesamum indicum | XP_011091785.1 | 1e-65 | 1.0012 |
| LjbZIP10 | KT218634 | transcription factor TGA1 isoform X2 | Vitis vinifera | XP_010652849.1 | 0.0 | 0.9983 |
| LjbZIP11 | KT218635 | G-box-binding factor 4-like isoform X1 | Citrus sinensis | XP_006473064.1 | 2e-84 | 0.9990 |

**Supplementary Table S6.** Sequence Information and RPKM values of bZIPs for EMSA analysis

| **Gene** | **sequence** | **length** | **Read** **num** | **Rpkm** | **Group** | **Gene** | **sequence** | **length** | **Read** **num** | **Rpkm** | **Group** |
| --- | --- | --- | --- | --- | --- | --- | --- | --- | --- | --- | --- |
| rLJbZIP4 | 34226 | 1216 | 20 | 61.4288 | A | LJbZIP11 | 182123 | 874 | 1 | 3.4399 | A |
| rLJbZIP18 | 135875 | 857 | 24 | 104.5938 | C | LJbZIP8 | 172294 | 978 | 15 | 46.1112 | C |
| rLJbZIP1 | 19989 | 1077 | 25 | 86.6961 | D | LJbZIP10 | 182084 | 1179 | 36 | 91.8001 | D |

**Supplementary Table S7.** The content of CGAs in transgenic tobacco

| Compounds | Content (μg/g) | | |
| --- | --- | --- | --- |
|  | WT | pCambia1305 | LjbZIP8 |
| Neochlorogenic acid | 239.4 ± 7.9 | 233.6 ± 6.8 | 191.7 ± 8.5^**^ |
| Chlorogenic acid | 1439.6 ± 65.1 | 1502.6 ± 49.6 | 1310.0 ± 38.2^*^ |
| Cryptochlorogenic acid | 307.2 ± 10.6 | 302.6 ± 10.1 | 265.9 ± 12.2^***^ |

The Pearson correlation coefficients are marked with *, * * and *** for the significance levels of 0.05, 0.01 and 0.001 respectively.
